# Reconstruction of Ultrafine MnS‐Induced Vacancy‐Rich Co_9_S_6.29_ Precatalysts in Mesoporous S‐Doped N‐Rich Hollow Carbon Nanotubes Enables Dynamic O‐Vacancy Cycling for High‐Performance Zn‐Air Batteries

**DOI:** 10.1002/advs.76208

**Published:** 2026-06-26

**Authors:** Debarani Devi Khumujam, Saleem Sidra, Ram Babu Ghising, Dong Won Kim, Jong Hui Choi, Gwanho Lee, Benzhi Wang, Hyung Mo Jeong, Sang‐Il Choi, Do Hwan Kim, Jeung Ku Kang

**Affiliations:** ^1^ Department of Materials Science and Engineering and NanoCentury Institute Korea Advanced Institute of Science and Technology (KAIST) Daejeon Republic of Korea; ^2^ Department of Energy Storage/Conversion Engineering Jeonbuk National University Jeonju Republic of Korea; ^3^ School of Materials Science and Engineering Yeungnam University Gyeongsan Republic of Korea; ^4^ Department of Chemistry and Green‐Nano Materials Research Center, Kyungpook National University Daegu Republic of Korea; ^5^ Department of Smart Fab. Technology Sungkyunkwan University Suwon Republic of Korea

**Keywords:** dynamic O‐vacancy cycling, mesoporous S‐doped N‐rich hollow carbon nanotubes, near‐theoretical energy density and robust cycling stability in Zn‐air batteries, operando and density functional theory analyses, reconstruction of ultrafine MnS‐induced vacancy‐rich Co_9_S_6_._29_ precatalysts

## Abstract

Zn‐air batteries (ZABs) hold great promise when the air cathode offers abundant active sites, fast kinetics, and high reversibility for oxygen reduction/evolution reactions (ORR/OER). Here, we report vacancy‐rich Co_9_S_6_._29_ precatalysts formed via ultrafine MnS‐induced lattice distortion in mesoporous S‐doped pyridinic/graphitic N‐rich hollow carbon nanotubes (CM@SNHCTs), synthesized through in situ sulfidation and Zn/melamine evaporation from polydopamine‐coated Mn–Co/Zn metal–organic frameworks. MnS‐induced Co_9_S_6_._29_ formation enables ∼fourfold higher capacitance than IrO_2_, while the SNHCT generates Zn/melamine‐evaporated mesoporous S‐doped pyridinic N‐rich channels for rapid ion transport and graphitic N‐rich networks for facile electron conduction. CM@SNHCT precatalysts reconstruct into CoOOH/CoO active phases, enabling O‐vacancy formation via OH^−^ desorption during ORR and bidentate O_2_ adsorption during OER, as well as O‐vacancy healing governed by OH^−^ adsorption and deprotonation, as confirmed by *operando* Raman spectroscopy and ^1^ ^8^O‐labeled differential electrochemical mass spectrometry. Density functional theory calculations reveal low Gibbs free‐energy barriers for oxygen intermediates after reconstruction, while strong CM‐SNHCT binding ensure structural stability. Consequently, CM@SNHCT outperforms Pt/C for ORR and IrO_2_ for OER with electrochemical stability over 5000 cycles. Moreover, the ZAB assembled with CM@SNHCT cathode and high‐capacity Zn anode achieves near‐theoretical energy density (993.8 Wh·kg^−^^1^) and stable voltages after 250 charge–discharge cycles, establishing a state‐of‐the‐art ZAB platform.

## Graphical abstract

We develop vacancy‐rich Co_9_S_6.29_ precatalysts formed via ultrafine MnS‐induced lattice distortion in mesoporous S‐doped N‐rich hollow carbon nanotubes (CM@SNHCTs). This CM@SNHCT precatalyst is reconstructed into catalytically‐active cathode phases that enable efficient oxygen reduction/evolution reactions (ORR/OER) through dynamic O‐vacancy formation and healing, while the SNHCT framework provides mesoporous pyridinic N‐rich channels for rapid mass transport, graphitic N‐rich networks for efficient electron conduction, and strong CM‐SNHCT bonding for structural stability. Benefiting from this synergy, the CM@SNHCT‐based ZAB achieves high energy density and robust cycling stability, outperforming the ZABs with state‐of‐the‐art other cathodes.

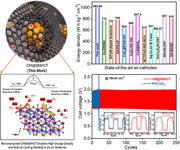

## 1 Introduction

Electrochemical energy storage systems (ESSs) that offer high energy density and robust cycling stability are essential for applications ranging from portable electronics to electric vehicles and grid‐scale storages [1, 2]. Lithium‐ion batteries (LIBs) have dominated the market for decades, but the energy density of LIBs is now approaching fundamental limits imposed by the finite capacity of intercalation‐based cathodes [3, 4]. This intrinsic limitation motivates the development of new alternative electrochemical energy storage technologies capable of delivering substantially higher energy densities. Metal–air batteries provide a promising next‐generation platform because they pair high‐capacity metal anodes with oxygen redox chemistry at the air cathode, effectively decoupling energy storage from cathode mass and enabling exceptionally high energy densities. Among them, Zn–air batteries (ZABs) are particularly attractive owing to the exceptionally high theoretical specific capacity of the Zn metal anode (820 Ah kg^−^^1^) [5, 6]. By utilizing atmospheric O_2_ as the active reactant at the air cathode, ZABs adopt an open‐cell configuration that enables a theoretical gravimetric energy density of 1086 Wh kg^−^^1^, far exceeding that of popular LIBs [7]. Energy storage in ZABs relies on oxygen reduction reaction (ORR) during discharge and oxygen evolution reaction (OER) during charging, eliminating the need to store oxidizing agents within the cell and substantially reducing system mass [8, 9, 10]. However, the practical realization of high‐performance ZABs remains severely constrained by poor activity and durability for ORR and OER under operating conditions. For example, the state‐of‐the‐art Pt/C+IrO_2_ catalyst, which is commonly adopted as the benchmark bifunctional air cathode, is limited by its inability to efficiently catalyze both ORR and OER within a single cathode framework. This limitation originates from the intrinsically distinct and often competing thermodynamic and kinetic requirements of ORR and OER. Specifically, the adsorption energies of oxygenated intermediates (^*^OH, ^*^O, and ^*^OOH) make it difficult for conventional catalysts to simultaneously optimize both ORR and OER pathways. Catalysts that are highly active for ORR often exhibit poor OER activity and vice versa, leading to compromised bifunctional efficiency and accelerated performance decay during charge–discharge cycling. Addressing these challenges necessitates the development of new bifunctional air‐cathode materials capable of regulating the adsorption and desorption energetics of oxygen intermediates, enabling efficient activity alongside robust cycling stability. Reducing catalyst particle size is an effective strategy to increase active‐site density and intrinsic activity, but the resulting high surface energy promotes aggregation during extended cycling, causing active‐site loss and performance decay [11]. Such particles inherently possess high surface energies, which thermodynamically favor aggregation under prolonged electrochemical cycling. Their morphological instability leads to a progressive loss of accessible active sites, deteriorates oxygen redox reversibility, and ultimately degrades both energy density and stability [12, 13, 14]. Beyond particle‐size effects, ZAB cathode performance is governed by dynamic phase reconstruction under electrochemical operation. During ORR/OER, cathodes undergo continuous structural and compositional transformations, with the initially synthesized phases acting as precatalysts rather than the true active species. Crucially, not all reconstructed phases are beneficial: irreversible reconstruction can generate unstable phases that accelerate catalyst degradation and performance loss. These support the necessity of rational precatalyst design strategies that not only stabilize active sites against aggregation, but also steer electrochemical reconstruction toward the formation of catalytically‐active cathode phases during ZAB operation. Such strategies are essential for developing efficient and durable air cathodes capable of delivering high energy density and robust cycling stability in ZABs.

In this work, we develop vacancy‐rich Co_9_S_6_._29_ precatalysts via ultrafine MnS‐induced lattice distortion in mesoporous S‐doped pyridinic/graphitic N‐rich hollow carbon nanotubes (CM@SNHCTs). Under electrochemical operating conditions, the as‐synthesized precatalysts undergo controlled in situ reconstruction into catalytically‐active oxygen reduction/evolution cathodes, enabling CM@SNHCT‐based ZABs to achieve near‐theoretical energy density while maintaining robust cycling stability. The synthesis strategy involves the growth of Co/Zn metal–organic frameworks (Co/Zn‐MOFs) on self‐assembled melamine trithiocyanurate (MTCA) nanorods, followed by Mn impregnation, polydopamine (PDA) coating, and subsequent in situ sulfidation accompanied by Zn/melamine evaporation. During sulfidation, ultrafine Co–Mn heterostructures are formed, significantly increasing the density of accessible catalytic sites. Simultaneously, ultrafine MnS incorporation induces pronounced lattice distortion at Co sites, which promotes the formation of vacancies and stabilizes a non‐stoichiometric Co_9_S_6_._29_ phase. The concurrent evaporation of Zn and melamine templates generates abundant mesopores within the SNHCT framework, facilitating rapid mass transport of reactants and intermediates while preserving structural integrity during repeated electrochemical cycling. MOFs, which offer abundant organic ligands and secondary building units (SBUs) for metal coordinations, are suited for heteroatom incorporation. The incorporation of Co and Mn ions into the SBUs of MOFs yields coordinatively unsaturated metal centers (CUMs), which can promote OH adsorption and accelerate deprotonation kinetics during oxygen electrocatalysis. However, such CUM sites lead to the excessively strong binding of ^*^O and ^*^OOH intermediates, which hampers ORR/OER reversibility and accelerates catalyst degradation. This limitation is addressed by S incorporation, which tunes the electronic structure of the metal centers. Owing to their electron‐withdrawing character, S species increase the positive charge density on Co sites and weaken overly strong oxygen adsorption, thereby tailoring the binding energetics of oxygenated intermediates to enable enhanced bifunctional catalytic activity. Moreover, *operando* experimental characterizations are combined with first‐principles atomistic simulations to identify the true catalytically active species, resolve transient reconstruction pathways, and quantitatively correlate their Gibbs free‐energies and atomic structures with catalytic activity and durability. MnS‐mediated Co_9_S_6_._29_ precatalysts are demonstrated to reconstruct into catalytically‐active hydroxide and oxide phases under operating conditions, while *operando* differential electrochemical mass spectrometry (DEMS) provides direct evidence for the involvement of a lattice oxygen mechanism (LOM) during oxygen electrocatalysis. Besides, the efficiency and reversibility of oxygen redox processes associated with the reconstructed cathodic phases are evaluated through extended discharge–charge cycling. Density functional theory (DFT) calculations are also performed to elucidate how electrochemical reconstruction modulates the Gibbs free‐energy barriers associated with key oxygen intermediates, and assess the formation energies and structural stability of CM@SNHCT. In addition, to evaluate the device‐level performance enabled by the CM@SNHCT air cathode, a full ZAB is assembled using a CM@SNHCT cathode and a Zn metal anode. The electrochemical characteristics of the resulting CM@SNHCT‐based ZAB, including energy density and cycling stability, are subsequently examined to assess how catalyst‐level activity and durability translate into practical battery performance. Additionally, to validate practical viability, multiple CM@SNHCT‐based ZABs are connected in series and evaluated for their ability to deliver sufficient output voltage and sustained power capable of driving a digital timer and a light‐emitting diode (LED).

## 2 Results and Discussion

**FIGURE 1:**
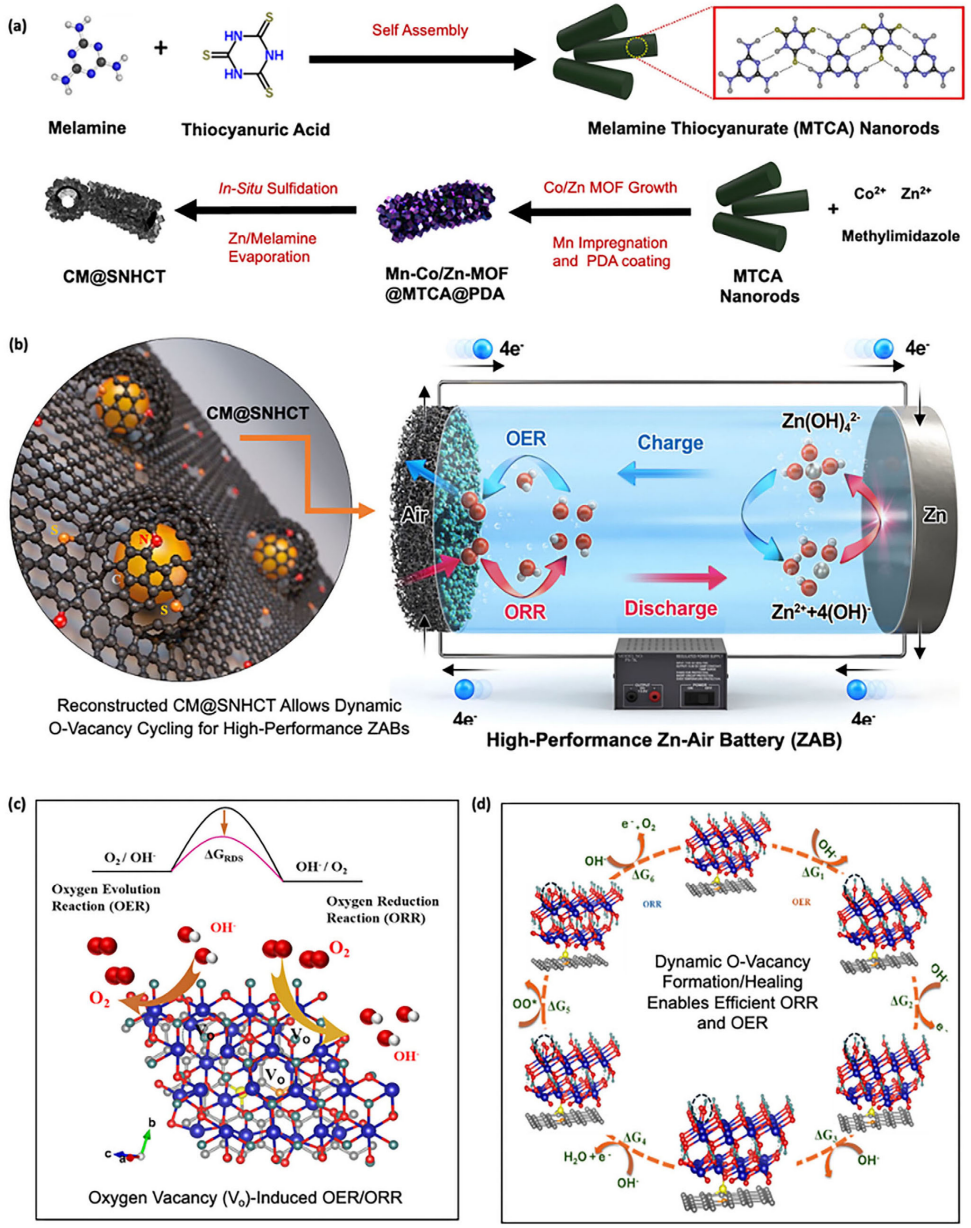
Design, structure, and oxygen electrocatalysis mechanism of CM@SNHCTs. (a) Stepwise synthesis of CM@SNHCTs. (b) Structural schematic of CM@SNHCT illustrating vacancy‐rich active phases reconstructed during electrochemical operation. (c) O vacancy‐induced mechanisms to reduce Gibbs free energy barriers for ORR/OER. (d) Dynamic lattice O‐vacancy formation and healing enabling efficient ORR and OER.

The synthetic strategy to construct the CM@SNHCT architecture is depicted in Figure 1a. One‐dimensional MTCA nanorods are first generated via supramolecular self‐assembly between melamine and thiocyanuric acid and serve as both structural templates and chemical precursors. Co/Zn‐MOFs are subsequently grown in situ on the MTCA nanorods, followed by Mn precursor impregnation and polydopamine (PDA) coating. The resulting PDA‐coated Mn–Co/Zn‐MOF@MTCA composites are then subjected to simultaneous in situ sulfidation and Zn/melamine evaporation, during which the MTCA framework supplies N and S heteroatoms, and the carbonized PDA layer forms a conductive carbon shell. Concurrent Zn/melamine evaporation generates interconnected mesopores that facilitate efficient mass transport. Figure 1b illustrates that CM@SNHCT undergoes electrochemical reconstruction under operating conditions, giving rise to O‐vacancy‐rich active phases for ORR/OER. As illustrated in Figure 1c, the reconstructed O vacancy‐rich active sites effectively lower the Gibbs free‐energy barriers associated with key oxygenated intermediates for ORR and OER, while the SNHCT framework provides continuous conductive pathways for rapid electron transport and interconnected Zn/melamine‐evaporated mesoporous channels for efficient ion transport. Strong interfacial bonding between the CM active domains and the SNHCT carbon matrix further stabilizes the ultrafine heterostructures against aggregation, suppressing structural degradation during prolonged electrochemical cycling and contributing to the overall durability of the cathode. Furthermore, the LOM enabled by dynamic O‐vacancy formation/healing is schematically depicted in Figure 1d. During OER, adsorption of OH^−^ species on Co sites activates lattice oxygen within the reconstructed CoOOH/CoO phases, promoting O–O bond formation through direct lattice‐oxygen participation and generating O vacancies as lattice oxygen is released. During subsequent ORR, these O vacancies are replenished through reincorporation of lattice oxygen, completing a reversible vacancy formation–healing cycle. This lattice‐oxygen participation in both OER and ORR indicates a dynamically coupled redox mechanism responsible for the high catalytic activity and reversibility of the CM@SNHCT cathode.

**FIGURE 2:**
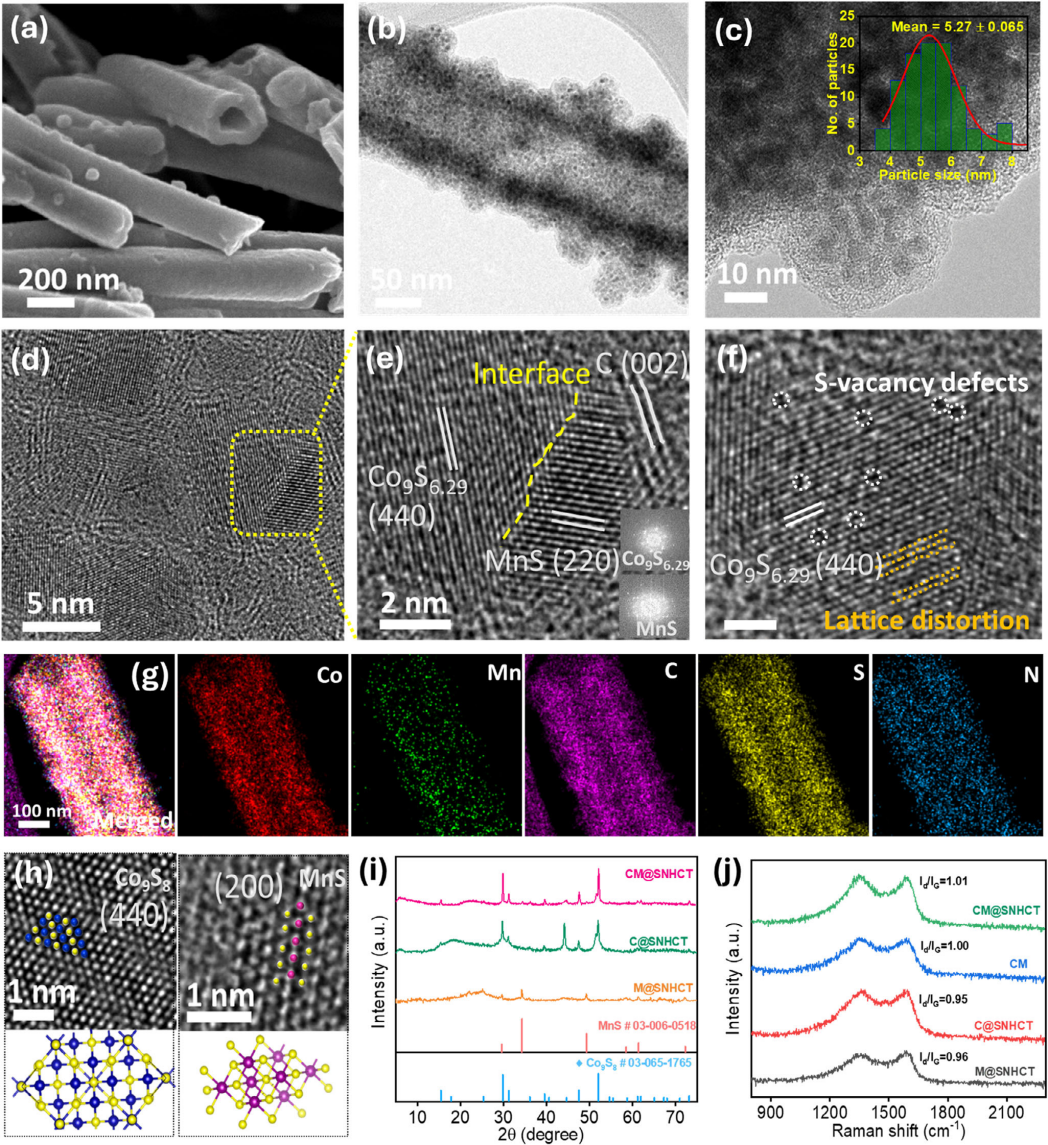
Structural characterizations of CM@SNHCT. (a) Scanning electron microscopy (SEM) image. (b) Transmission electron microscopy (TEM) image. (c, d) High‐resolution TEM (HRTEM) images. (e) HRTEM image of the CM heterostructure interface with the corresponding fast Fourier transform (FFT) patterns (inset). (f) HRTEM image of a Co sulfide phase showing lattice distortion and S‐vacancy defects. (g) TEM energy‐dispersive X‐ray spectroscopy (EDS) elemental mapping images. (h) Atomic‐resolution HRTEM images of Co sulfide and Mn sulfide (200) planes in CM@SNHCT. (i) X‐ray diffraction (XRD) patterns and (j) Raman spectra of M@SNHCT, C@SNHCT, CM, and CM@SNHCT.

The morphological and structural characteristics of CM@SNHCT were also investigated using scanning/transmission electron microscopies (SEM/TEM). As exhibited in the SEM and TEM images in Figure 2a,b and Figure S1, the CM@SNHCT exhibits well‐defined MOF‐derived heterostructures composed of bimetallic sulfides uniformly embedded within an SHCNT. Notably, the tubular morphology of C@SNHCT remains well preserved after Mn impregnation, indicating excellent structural robustness during the post‐synthetic modification process. In contrast, when the PDA coating step was omitted, the pyrolyzed product corresponding to the CM sample exhibited a polyhedral morphology rather than a tubular architecture, as shown in Figure S2a. This observation highlights the critical role of the PDA coating in directing and stabilizing the formation of hollow carbon tube structures during thermal conversion. Energy‐dispersive X‐ray spectroscopy (EDS) elemental mapping based on TEM measurements (Figure S2b,c) confirms the homogeneous distribution of Co, Mn, C, S, and N throughout the CM@SNHCT architecture, verifying the successful formation of Co–Mn sulfide heterostructures embedded within a N‐doped carbon matrix. High‐resolution TEM (HR‐TEM) images (Figure 2c) further reveal that ultrafine nanoparticles are uniformly dispersed within the graphitized carbon framework, with no detectable aggregation or clustering of metal species. The inset presents a statistical size distribution analysis, indicating an average particle size of ∼5.27 nm for the CM heterostructures. Such small particles significantly enhance surface exposure and maximize the number of accessible active sites for oxygen‐intermediate adsorption and desorption during both OER and ORR. Further HR‐TEM analysis (Figure 2d,e) provides detailed insight into the crystallographic features of the heterostructures formed within the SNHCT framework. Lattice fringes with interplanar spacings of 0.352 nm correspond to the (002) plane of graphitized carbon, while spacings of 0.175 and 0.185 nm can be assigned to the (440) plane of Co_9_S_6_._29_ (JCPDS No. 065–1765) and the (220) plane of MnS (JCPDS No. 006–0518), respectively. Importantly, the atomic‐scale interface between the Co_9_S_6_._29_ (440) and MnS (220) planes, highlighted by a yellow dotted line in Figure 2e, serves as a highly active region for the facile adsorption, activation, and conversion of oxygen intermediates. In addition, pronounced lattice distortions are observed within the Co_9_S_6_._29_ domains of CM@SNHCT (Figure 2f). Specifically, Co^2^^+^ ions (t_2g_^6^e_g_^1^) exhibit a strong Jahn–Teller effects [15] due to asymmetric occupancy of the e_g_ orbitals, whereas Mn^2^^+^ ions (t_2_g^5^e_g_^0^) display a weaker distortion associated with uneven t_2g_ orbital occupancy. These cooperative distortions induce local lattice strain and are confirmed to promote the formation of vacancy defects. The presence of numerous hole‐like features in the HR‐TEM images provides direct evidence for the generation of vacancies within the sulfide lattice. High‐angle annular dark‐field scanning TEM (HAADF‐STEM) imaging combined with EDS elemental mapping (Figure 2g) further verifies the uniform spatial distribution of Co, Mn, C, S, and N within the CM@SNHCT structure. Quantitative elemental analysis derived from the EDS spectrum (Table S1) reveals an atomic S/Co ratio of 0.699, which is significantly lower than the theoretical value of 0.889, confirming the presence of abundant S vacancies. Atomic‐resolution HR‐TEM images of the Co and Mn sulfide phases (Figure 2h) offer additional insights into the structural integration and coherent interfacial coupling between the two sulfide components. To evaluate phase composition and crystallinity, X‐ray diffraction (XRD) analysis was performed (Figure 2i). For CM@SNHCT, C@SNHCT, M@SNHCT, and CM, characteristic diffraction peaks at approximately 25° and 44° are observed, corresponding to the (002) and (101) planes of graphitized carbon, respectively. In CM@SNHCT, C@SNHCT, and CM, additional diffraction peaks located at 15.5°, 29.8°, 31.3°, 39.6°, 47.6°, and 52.1° can be indexed to the (111), (311), (222), (331), (511), and (440) planes of cubic Co_9_S_8_. Moreover, CM@SNHCT exhibits distinct peaks at 34.5° and 39.6°, corresponding to the (200) and (220) planes of MnS, respectively, confirming the coexistence of Co–Mn heterostructured sulfide phases. The absence of diffraction peaks associated with metallic Co or Mn suggests that both metals were fully converted into sulfide phases during in situ sulfidation, with no formation of metallic clusters. In addition, Raman spectroscopy was used to probe the carbon structure within the catalysts (Figure 2j). Two characteristic bands are observed at approximately 1350 cm^−^^1^ (D band) and 1590 cm^−^^1^ (G band), corresponding to defective and graphitic carbon, respectively. CM@SNHCT and CM exhibit higher intensity ratios of the D band to the G band, with I_D_/I_G_ values of 1.01 and 1.00, respectively, compared to C@SNHCT (I_D_/I_G_ = 0.95) and M@SNHCT (I_D_/I_G_ = 0.96). These results indicate an increased density of structural defects. Additionally, the pore structures of the catalysts were analyzed using N_2_ adsorption–desorption measurements (Figure S3a). All samples display type IV isotherms with pronounced hysteresis loops, indicative of hierarchical porous architectures. A sharp N_2_ uptake at low relative pressures (P/P_0_< 0.1) suggests the presence of abundant micropores, while the steep increase at high relative pressures (P/P_0_> 0.9) indicates the coexistence of mesopores. Micropores contribute to enhanced catalytic activity by providing high‐density active sites, whereas mesopores facilitate efficient mass transport of reactants and products [16, 17]. Brunauer–Emmett–Teller (BET) measurements show the specific surface areas of 300.12 m^2^·g^−^^1^ for CM@SNHCT, 195.88 m^2^·g^−^^1^ for CM, 176.45 m^2^·g^−^^1^ for C@SNHCT, and 164.19 m^2^·g^−^^1^ for M@SNHCT, in which the high surface area of CM@SNHCT is ascribed to Zn/melamine evaporation–induced mesopore formation, S vacancy generation arising from Co sulfide lattice distortion, and ultrafine MnS formation via in situ sulfidation. Additionally, Barrett–Joyner–Halenda (BJH) analysis (Figure S3b) confirms the coexistence of micropores and mesopores.

**FIGURE 3:**
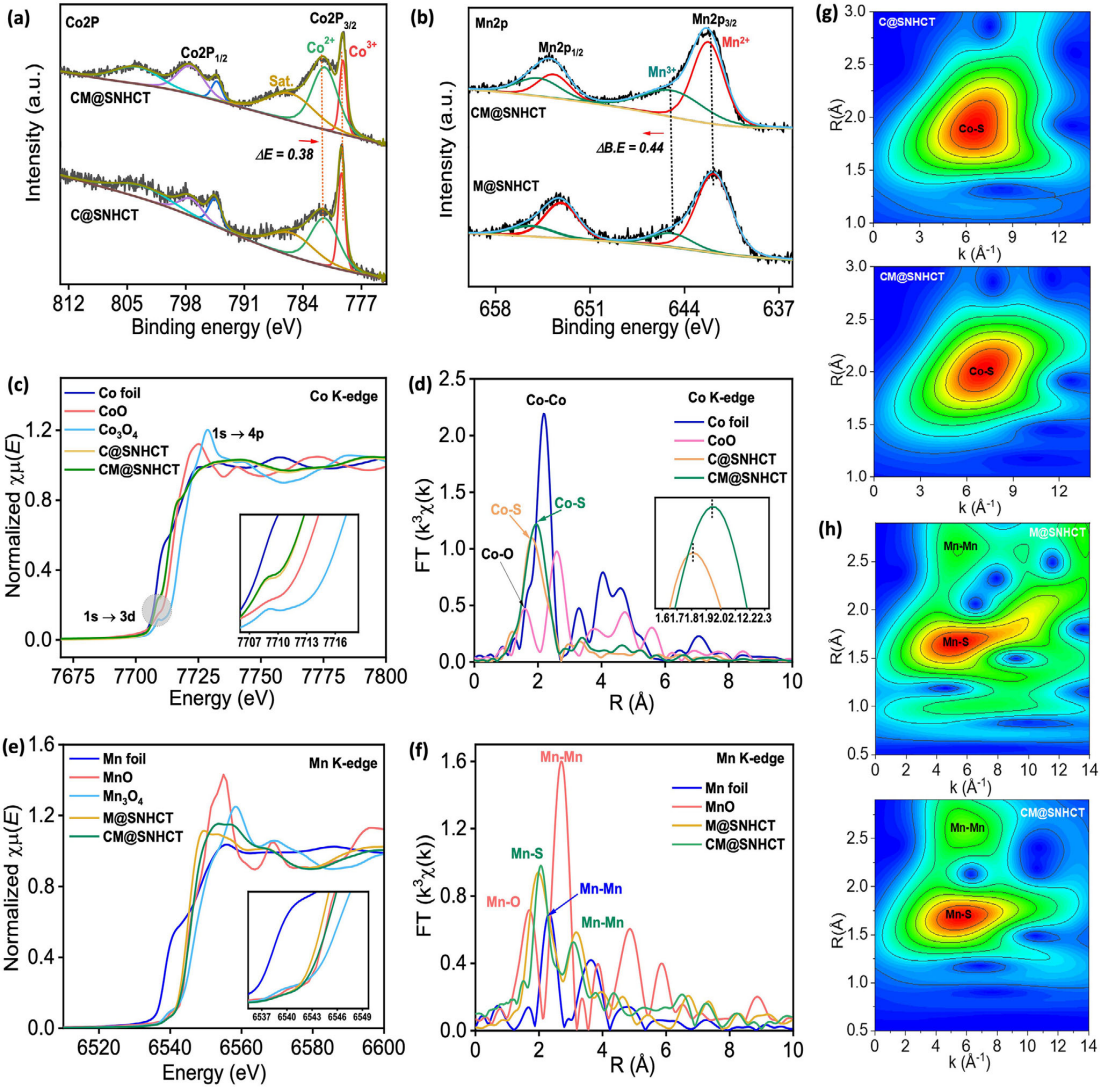
Material characterizations of CM@SNHCT. High‐resolution X‐ray photoelectron spectroscopy (XPS) spectra for (a) Co2p and (b) Mn2p of CM@SNHCT. (c) Co K‐edge XANES spectra and (d) FT extended X‐ray absorption fine structure (FT‐EXAFS) spectra of CM@SNHCT, C@SNHCT, Co_3_O_4_, CoO, and Co foil. (g) Wavelet transforms (WTs) of CM@SNHCT and C@SNHCT. (e) Mn K‐edge X‐ray absorption near edge spectroscopy (XANES) spectra and (f) FT‐EXAFS spectra of CM@SNHCT, C@SNHCT, Co_3_O_4_, CoO, and Co foil. (h) WTs of CM@SNHCT and M@SNHCT.

The surface chemical states and ionic configurations of the catalysts were further investigated to elucidate their electronic structure and active‐site chemistry. X‐ray photoelectron spectroscopy (XPS) spectra revealed the presence of Co 2p, C 1s, N 1s, and S 2p signals in C@SNHCT, with an additional Mn 2p signal detected in CM@SNHCT, demonstrating the successful incorporation of Mn into the heterostructured framework (Figure S4a). A weak O 1s peak was also observed, attributed to slight surface oxidation upon exposure to air. To gain deeper insight into charge redistribution and interfacial electronic coupling at the active sites, high‐resolution XPS spectra of Co 2p, Mn 2p, C 1s, N 1s, and S 2p were systematically deconvoluted. The high‐resolution Co 2p spectrum (Figure 3a) reveals two characteristic spin–orbit doublets corresponding to Co 2p_3_/_2_ and Co 2p_1_/_2_ at binding energies of 780.6 and 796.5 eV, respectively, accompanied by satellite peaks at 785.6 and 802.5 eV. Deconvolution of the Co 2p spectrum for CM@SNHCT reveals Co^3^^+^ species at 778.3 and 793.2 eV and Co^2^^+^ species at 780.9 and 796.95 eV. Notably, a negative binding energy shift of approximately 0.38 eV is observed for Co 2p in CM@SNHCT relative to C@SNHCT, indicating electron enrichment at the Co sites induced by heterostructure formation and interfacial charge transfer [18]. Correspondingly, the Co^3^^+^/Co^2^^+^ ratio decreases markedly from 0.59 in C@SNHCT to 0.30 in CM@SNHCT, confirming the partial reduction of Co to a lower oxidation state, which is beneficial for enhancing catalytic activity. Similarly, the high‐resolution Mn 2p spectrum of CM@SNHCT (Figure 3b) displays two prominent peaks at 641.3 and 653.2 eV, assigned to Mn 2p_3_/_2_ and Mn 2p_1_/_2_, respectively. Compared to M@SNHCT, the Mn 2p peaks in CM@SNHCT shift toward higher binding energies, suggesting electron depletion at the Mn sites. Quantitative fitting reveals that the Mn^3^^+^/Mn^2^^+^ ratio increases from 0.26 in M@SNHCT to 0.41 in CM@SNHCT, indicating partial oxidation of Mn^2^^+^ to Mn^3^^+^. This oxidation process enhances electrocatalytic activity by strengthening Lewis acid–base interactions with oxygenated intermediates during ORR and OER [19, 20], indicating interfacial electron transfer from Mn sulfide to Co sulfide within the CM heterostructure. The deconvoluted C 1s spectra (Figure S4b) consist of four distinct components located at 283.9 eV (C = C–S), 284.8 eV (C–S), 285.4 eV (C–N), and 286.6 eV (C–O). Such heteroatom doping modulates the electronic structure of carbon, improving conductivity and interfacial charge transport. The high‐resolution N 1s spectra (Figure S4c) can be fitted into pyridinic N (398.5 eV), metal–nitrogen (M–N, 399.2 eV), and graphitic N (400.1 eV) species in both C@SNHCT and CM@SNHCT. Notably, pyrrolic N is absent, indicating its complete conversion into more thermodynamically stable pyridinic and graphitic N species during high‐temperature pyrolysis [21]. These N configurations signal the donation of electrons to adjacent sp^2^‐hybridized carbon atoms, facilitating electron transfer and accelerating reaction kinetics for both ORR and OER [22]. Analysis of the S 2p spectra (Figure S4d) reveals two dominant peaks at 161.6 and 162.8 eV, corresponding to S^2^^−^ species coordinated with Co and Mn atoms in CM@SNHCT, C@SNHCT, and M@SNHCT. In addition, higher binding energy components at 163.8 eV (C–S–C), 164.8 eV (C = S), and 168.5 eV (C–SO_x_–C) confirm the incorporation of S atoms into the carbon framework. Compared with C@SNHCT and M@SNHCT, the S 2p peaks of CM@SNHCT exhibit a noticeable shift toward lower binding energies, indicating CM heterostructure formation. Notably, the presence of hydrophobic C–S–C motifs is expected to facilitate mass transport and gas diffusion within cathode electrodes [23]. Additionally, surface wettability was evaluated using water contact angle measurements (Figure S5). The contact angle increases dramatically from 37.11° for MOF‐derived Co–N/C (prepared without the MTCA substrate and Mn precursor) to 90.98° for C@SNHCT and further to 121.31° for CM@SNHCT. This enhanced hydrophobicity is attributed to sulfur doping and the formation of bimetallic sulfide phases, as corroborated by XPS analysis. Moreover, to probe the chemical states and coordination environments of the metal centers, X‐ray absorption spectroscopy (XAS) was conducted. Co K‐edge X‐ray absorption near‐edge structure (XANES) spectra (Figure 3c) reveal that the absorption edge positions of both CM@SNHCT and C@SNHCT are significantly higher than that of Co foil but lower than that of CoO, indicating an average Co valence state close to +2. Importantly, CM@SNHCT exhibits a slight negative shift in edge position relative to C@SNHCT, consistent with electron transfer from Mn sulfide to Co sulfide, in good agreement with XPS results. The presence of pronounced pre‐edge features further suggests local symmetry distortion around Co sites, confirming the formation of vacancy defects. Fourier‐transform extended X‐ray absorption fine structure (FT‐EXAFS) analysis in R‐space (Figure 3d) reveals a dominant peak at approximately 1.93 Å, corresponding to Co–S coordination in CM@SNHCT. This bond length is longer than the Co–S distance observed in C@SNHCT (1.78 Å), indicating weakened metal–oxygen binding strength, which is beneficial for enhancing catalytic stability and reaction kinetics. Notably, no Co–Co scattering peaks are detected in either CM@SNHCT or C@SNHCT, confirming the absence of metallic Co clusters. The increased Co–S peak intensity in CM@SNHCT suggests reduced structural disorder due to Mn incorporation. Mn K‐edge XANES spectra of CM@SNHCT and M@SNHCT, together with Mn_3_O_4_, MnO, and Mn foil references, are presented in Figure 3e. Both CM@SNHCT and M@SNHCT exhibit edge positions indicative of a Mn valence state close to +2. However, a subtle positive shift in the Mn K‐edge of CM@SNHCT relative to M@SNHCT suggests partial oxidation of Mn^2^^+^ to Mn^2^^+^^δ^, supporting the occurrence of interfacial charge redistribution. This electron transfer effectively modulates the electronic structure of Co sites, thereby promoting high catalytic activity. FT‐EXAFS analysis (Figure 3f) reveals two characteristic peaks at approximately 1.93 and 2.82 Å, corresponding to Mn–S and Mn–Mn coordination, respectively. Furthermore, wavelet transform (WT) analysis was employed to gain deeper insight into the local atomic coordination environments. The WT contour plots (Figure 3g) display intensity maxima at 6.5 and 6.8 Å^−^^1^ in k‐space for CM@SNHCT and C@SNHCT, respectively, which are markedly lower than those observed for CoO (7.2 Å^−^^1^) and Co foil (8.25 Å^−^^1^), as shown in Figure S6a These shifts indicate that cobalt predominantly exists in a Co–S coordination environment rather than Co–O or Co–Co bonding. The WT contour plots for Mn (Figure 3h) show a maximum intensity at 4.9 Å^−^^1^ for CM@SNHCT, which lies between those for MnO (4.4 Å^−^^1^) and Mn foil (5.6 Å^−^^1^) in Figure S6b, validating the Mn–S bonding environment.

**FIGURE 4:**
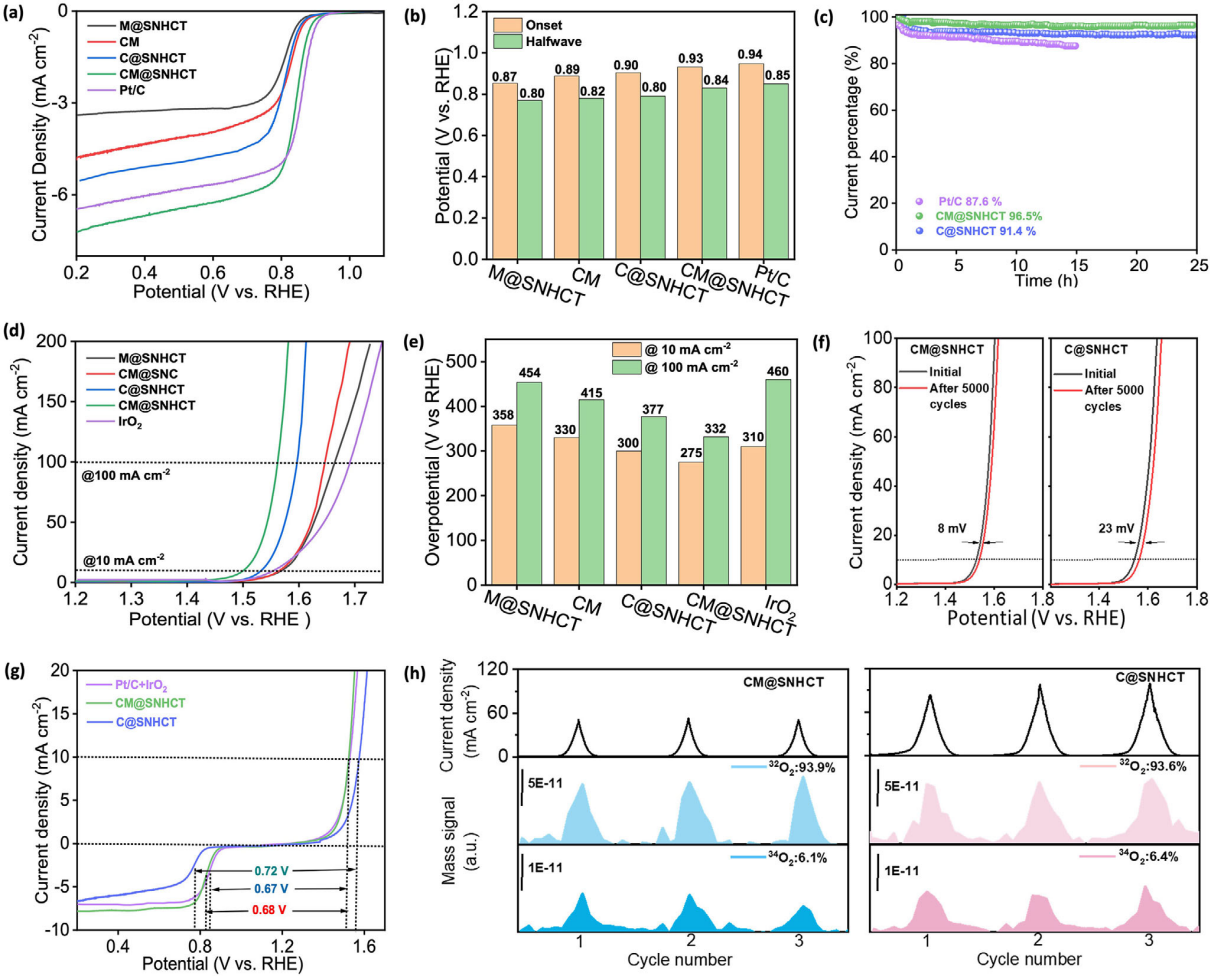
ORR/OER performance characterizations of the CM@SNHCT cathode. (a) linear sweep voltammetry (LSV) curves and (b) onset and half‐wave potentials of M@SNHCT, CM, C@SNHCT, CM@SNHCT, and Pt/C for ORR. (c) Comparison of chronoamperometric responses of CM@SNHCT, C@SNHT, and Pt/C. (d)) LSV curves, (e) overpotentials of M@SNHCT, CM, C@SNHCT, CM@SNHCT, and IrO_2_ for OER. (f) OER LSV curves of CM@SNHCT and C@SNHCT before the test and after 5000 cycles. (g) Overall polarization curves of CM@SNHCT, C@SNHCT, and Pt/C+IrO_2_. (h) DEMS results by ^18^O‐isotopic labeling experiments for CM@SNHCT and C@SNHCT.

To evaluate the ORR activity of the prepared catalysts, cyclic voltammetry (CV) and linear sweep voltammetry (LSV) measurements were performed using a rotating disk electrode (RDE) in O_2_‐saturated 0.1 m KOH under a standard three‐electrode configuration. The CV curves for CM@SNHCT are recorded at a scan rate of 5 mV·s^−^^1^, as shown in Figure S7a. A pronounced cathodic peak at 0.79 V vs. the reversible hydrogen electrode (RHE) was observed in O_2_‐saturated electrolyte, whereas no such feature appeared under N_2_, indicating excellent ORR activity. The ORR polarization curves (Figure 4a,b) reveal that CM@SNHCT delivers an onset potential (E_o_) of 0.93 V and a half‐wave potential (E_1_/_2_) of 0.84 V, which are comparable to those of commercial Pt/C (E_o_ = 0.94 V, E_1_/_2_ = 0.85 V). Notably, CM@SNHCT exhibits a higher diffusion‐limiting current density than Pt/C, demonstrating superior ORR kinetics. The observed limiting current exceeds ∼6 mA·cm^−^^2^, attributed to the enlarged effective active surface area and enhanced local O_2_ concentration near the electrode surface [24]. In addition, CM@SNHCT displays superior methanol tolerance compared to Pt/C (Figure S7b). In contrast, single‐metal sulfide counterparts exhibit inferior ORR performance. M@SNHCT shows an E_o_ of 0.87 V and E_1_/_2_ of 0.80 V, while C@SNHCT delivers an E_o_ of 0.90 V and E_1_/_2_ of 0.80 V. The catalyst prepared without PDA coating exhibits moderate activity (E_o_ = 0.89 V, E_1_/_2_ = 0.82 V) but suffers from a significantly lower diffusion‐limiting current, which is attributed to limited exposure of active sites. Koutecky–Levich (K–L) analysis derived from LSV curves at different rotation speeds (Figure S8 indicates an electron transfer number (n) close to 4 for all catalysts, confirming a dominant four‐electron ORR pathway. Chronoamperometric stability tests (Figure 4c) demonstrate that CM@SNHCT retains 96.5% of its initial current density after 25 h of continuous operation, outperforming C@SNHCT (91.4%). In contrast, Pt/C exhibits rapid degradation, maintaining only 87.8% of its initial current density after 15 h. These results highlight the superior durability of CM@SNHCT under alkaline ORR conditions. The superior catalytic activity and durability of CM@SNHCT over the control samples are attributed to the optimized hydrophobicity that facilitates oxygen diffusion and stabilizes the gas‐liquid‐solid triple phase interface by allowing optimum electrolyte penetration and preventing electrolyte flooding within the porous catalysts’ framework. This leads to maintaining continuous gas transport pathways and preserves the accessibility of the catalytically active sites during prolonged operation, thereby promoting faster oxygen transport kinetics and enhanced electrochemical durability. Further, OER activity was evaluated by LSV in 1 m KOH with 95% iR correction (Figure 4d). CM@SNHCT requires low overpotentials of 275 mV at 10 mA·cm^−^^2^ and 332 mV at 100 mA·cm^−^^2^, outperforming all comparison catalysts, including commercial IrO_2_, as shown in Figure 4e and Figure S9. Furthermore, CM@SNHCT exhibits the smallest Tafel slope of 64.4 mV·dec^−^^1^, indicating favorable OER kinetics. Electrochemical double‐layer capacitance (C_dl_) measurements (Figure S10) reveal a high C_dl_ value of 40.62 mF·cm^−^^2^ for CM@SNHCT, which is about fourfold higher than that of IrO_2_, indicating substantially increased electrochemically active surface area. Long‐term cycling stability was also evaluated by repeated LSV cycling (Figure 4f). CM@SNHCT shows negligible performance degradation, with only an 8 mV increase in overpotential after 5000 cycles at 10 mA·cm^−^^2^, whereas C@SNHCT exhibits a significantly larger increase of 23 mV under identical conditions. To assess bifunctional reversibility, the potential difference (ΔE) between the ORR E_1_/_2_ and the OER potential at 10 mA·cm^−^^2^ was calculated. CM@SNHCT delivers a ΔE of 0.68 V, comparable to the benchmark Pt/C+IrO_2_ system (0.67 V) and superior to C@SNHCT (0.72 V), indicating excellent ORR/OER reversibility (Figure 4g). Performance comparisons with recently reported bifunctional catalysts are summarized in Table S2. To elucidate the origin of the enhanced OER activity and identify the true active phases, *operando* Raman spectroscopy was performed (Figure S11). For C@SNHCT, structural reconstruction occurs immediately upon immersion in alkaline electrolyte, with complete conversion to CoO_2_ at 1.45 V vs. RHE, which remains as the stable but catalytically inferior OER phase. In contrast, CM@SNHCT undergoes reconstruction at a lower potential of 1.35 V, accompanied by the emergence of Raman bands at ∼485 and 515 cm^−^^1^ corresponding to Co–O bending and stretching modes in CoOOH. Simultaneously, the Co–S vibration near 670 cm^−^^1^ gradually blue‐shifts, indicating the progressive transformation of Co–S into Co–O species. These observations confirm that CoOOH/CoO phases constitute the true OER‐active species. CoO_2_, characterized by a half‐filled e_g_ orbital, stabilizes superoxo (OO^−^) intermediates and hinders O_2_ desorption, resulting in poor OER kinetics [25, 26]. Notably, no Raman signals corresponding to Mn‐based oxides were detected during operation, indicating that MnS does not act as a primary redox center. Instead, MnS electronically modulates neighboring Co sites, suppressing overoxidation to inactive CoO_2_ and stabilizing catalytically active CoOOH/CoO phases. This synergistic modulation accounts for the superior OER performance of CM@SNHCT. The OER mechanism was further investigated by evaluating activity across KOH concentrations from pH 14 to 13.5. Both CM@SNHCT and C@SNHCT exhibit similar decreases in activity upon electrolyte dilution (Figure S12), consistent with a lattice oxygen mechanism (LOM), in which proton and electron transfer steps are decoupled and strongly pH dependent [27]. Additional validation was obtained using tetramethylammonium hydroxide (TMAOH) as a chemical probe. The positively charged TMA^+^ cations competitively bind lattice oxygen species, suppressing LOM kinetics. Both catalysts show significant activity loss in 1 m TMAOH, further supporting lattice oxygen participation. Direct evidence for LOM was obtained via *operando* DEMS with ^1^ ^8^O isotope labeling. After electrochemical labeling in H_2_^1^ ^8^O‐containing KOH, catalysts were tested in H_2_^1^ ^6^O electrolyte while monitoring ^3^ ^2^O_2_ and ^3^ ^4^O_2_ evolution during OER. As shown in Figure 4h, both CM@SNHCT and C@SNHCT exhibit clear ^3^ ^4^O_2_ signals, confirming lattice oxygen exchange. Quantitative analysis yields ^3^ ^2^O_2_:^3^ ^4^O_2_ ratios of 93.9:6.1 for CM@SNHCT and 93.6:6.4 for C@SNHCT, which far exceed the natural abundance of ^1^ ^8^O (0.2%) [28, 29]. These results validate the LOM pathway [30], in which vacancy formation/healing cycle reduces O^*^ binding energy and facilitates efficient bifunctional catalysis. CM@SNHCT exhibits markedly superior activity due to MnS‐mediated stabilization of the reconstructed CoOOH/CoO phases, enabling dynamic O‐vacancy formation/healing in which dynamic O‐vacancy formation occurs through OH^−^ desorption during ORR or bidentate oxygen adsorption during OER, and O‐vacancy healing proceeds via OH^−^ adsorption and subsequent deprotonation. This reversible vacancy formation–healing cycle reduces O^*^ binding energy and facilitates efficient bifunctional catalysis via the LOM pathway. In contrast, C@SNHCT undergoes excessive oxidation to CoO_2_, leading to the formation of strongly bound Co^4^^+^–O species that suppress oxygen‐vacancy healing. As a result, the superior bifunctional ORR/OER performance of CM@SNHCT is attributed to the combined effects of regulated surface wettability, heterointerfaces, MnS‐stabilized reconstructed phases, and dynamically regulated lattice oxygen vacancies.

**FIGURE 5:**
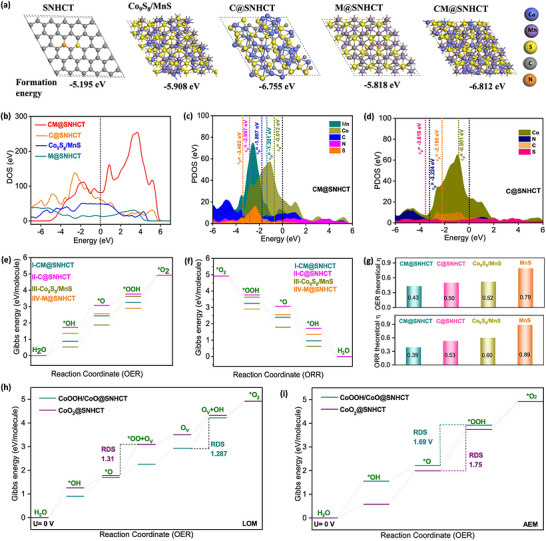
Atomic/electronic structures and proposed ORR/OER mechanisms of CM@SNHCT as the cathode. (a) Structural models of SNHCT, Co_9_S_6.29_/MnS, C@SNHCT, M@SNHCT, and CM@SNHCT without their corresponding formation energies. (b) density of states (DOS) plots for Co_9_S_6.29_/MnS, C@SNHCT, M@SNHCT, and CM@SNHCT. Projected DOS plots for both d orbitals of Co and Mn and also p orbitals of C, S, and N for (c) CM@SNHCT and (d) C@SNHCT. Calculated Gibbs free energy diagrams for (e) OER and (f) ORR, and (g) theoretical overpotential values for OER and ORR of CM@SNHCT, C@SNHCT, Co_9_S_8_/MnS, and MnS. Gibbs free energy diagrams of OER for (h) lattice oxygen mechanism and (i) adsorbate‐evolution mechanism pathways for CoOOH/CoO@SNHCT and CoO_2_@SNHCT.

To complement the experimental observations and provide atomistic insight into the role of Mn in tuning the electronic structure and reaction kinetics, DFT calculations were performed. The optimized geometries of individual precatalysts and interfacial heterostructure models were constructed based on lattice‐matched crystallographic planes, as shown in Figure 5a and Figure S13. Among all evaluated models, CM@SNHCT exhibits the most negative formation energy (–6.81 eV), exceeding those of C@SNHCT (–6.76 eV), M@SNHCT (–5.82 eV), Co_9_S_6_._29_/MnS (–5.91 eV), and pristine SNHCT (–5.20 eV). Because a more negative formation energy corresponds to higher thermodynamic stability, these results indicate that CM@SNHCT is the most stable configuration, consistent with its experimentally observed structural robustness. To elucidate electronic modulation at the atomic level, density of states (DOS) calculations were conducted for all models. As shown in Figure 5b, the DOS of each structure crosses the Fermi level (E_F_), confirming their metallic character and favorable electron‐transfer capability [31, 32]. A pronounced DOS modification is observed for C@SNHCT, arising from strong orbital overlap between Co 3d states and the surrounding S/N/C p orbitals, indicative of strong electronic coupling between cobalt sulfides and the heteroatom‐doped hollow carbon matrix. Upon formation of the CM heterostructure, further DOS redistribution occurs, reflecting additional interfacial electronic interactions. Because catalyst–intermediate interactions are governed by unoccupied electronic states, the d‐band centers (E_d_) of Co and Mn and the p‐band centers (E_p_) of S, N, and C were extracted from the projected DOS (PDOS), as shown in Figure 5c,d and Figure S14. The E_d_ of Co 3d shifts upward from –1.251 eV in Co_9_S_8_/MnS to –0.901 eV in C@SNHCT, indicating reduced filling of antibonding states due to carbon confinement. Following heterostructure formation, the Co E_d_ slightly downshifts to –0.912 eV in CM@SNHCT. On the other hand, the Mn E_d_ exhibits an upward trend in the sequence M@SNHCT < Co_9_S_8_/MnS < CM@SNHCT. A higher E_d_ relative to E_F_ implies stronger adsorption of oxygen intermediates due to reduced antibonding‐state occupancy, whereas a lower E_d_ suggests weaker binding [33]. For optimal bifunctional catalysis, E_d_ must be precisely balanced to enable efficient adsorption and desorption of ^*^OH, ^*^O, and ^*^OOH intermediates. These results demonstrate that S, N‐doped carbon confinement reduces excessive antibonding‐state filling at Co sites, while heterostructure formation further fine‐tunes Co E_d_ to an optimal regime for both ORR and OER. To evaluate reaction thermodynamics, Gibbs free‐energy changes (ΔG) for elementary steps along the four‐electron ORR and OER pathways were calculated at different active sites. The resulting free‐energy diagrams in Figure 5e–g and Figures S15 and S16 show uphill profiles for OER and downhill profiles for ORR. For CM@SNHCT, the rate‐determining step (RDS) for OER is the decomposition of OOH^*^ to release O_2_, whereas OH^*^ desorption constitutes the RDS for ORR. The calculated overpotentials are 0.43 V for OER at Co sites and 0.39 V for ORR at Mn sites. Correspondingly, CM@SNHCT exhibits the lowest ΔG values among all models, with 1.66 eV for OER and –0.84 eV for ORR, outperforming Co_9_S_6_._29_ (2.99 eV, 0.26 eV), MnS (3.17 eV, 0.31 eV), C@SNHCT (1.73 eV, –0.70 eV), M@SNHCT (2.02 eV, –0.34 eV), and Co_9_S_6_._29_/MnS (1.75 eV, –0.63 eV). Detailed ΔG values and overpotentials for all active sites are summarized in Tables S3 and S4. These results confirm that integrating a highly efficient bifunctional heterostructure within a heteroatom‐doped hollow carbon matrix substantially lowers oxygen‐intermediate adsorption/desorption barriers, accounting for the superior ORR/OER activity of CM@SNHCT. To elucidate the atomistic origin of reconstructed‐phase activity, DFT calculations were further performed for CoOOH/CoO@SNHCT and CoO_2_@SNHCT, as shown in Figure S17. The reconstructed structures, enriched with oxygen defects, can facilitate OH^−^ adsorption and deprotonation to promote O_2_ desorption via the adsorbate evolution mechanism (AEM), or alternatively enhance metal–oxygen covalency to activate lattice oxygen and enable the LOM [34, 35]. PDOS analysis (Figure S18) reveals that CoOOH/CoO@SNHCT exhibits a downward shift of the Fermi level and increased orbital overlap between Co 3d and O 2p states relative to CoO_2_@SNHCT, indicating stronger hybridization. This enhanced metal–oxygen covalency weakens Co–O bonds, facilitating lattice‐oxygen release during OER. The calculated Gibbs free‐energy profiles further corroborate experimental evidence for lattice‐oxygen participation. Along the LOM pathway (Figure 5h), both reconstructed catalysts display accessible reaction energetics, with RDS barriers of 1.31 eV for CoO_2_@SNHCT and 1.287 eV for CoOOH/CoO@SNHCT. In contrast, the conventional AEM pathway (Figure 5i) exhibits substantially higher energy barriers of 1.75 and 1.69 eV, respectively, indicating that AEM is energetically unfavorable. For comparison, MnO@SNHCT was also evaluated under both pathways, yielding higher overpotentials of 0.51 eV (LOM) and 0.54 eV (AEM) in Figure S19. The LOM pathway involves OH^−^ adsorption on Co sites, which activates lattice oxygen and promotes O–O coupling through direct lattice‐oxygen participation in CoOOH/CoO@SNHCT. This process provides direct evidence of a dynamic oxygen‐vacancy formation/healing cycle, in which lattice oxygen is extracted during OER and replenished during ORR. The adsorbate evolution mechanism (AEM) involving ^*^OH, O, and OOH intermediates is illustrated in Figure S20, while comparative reaction pathways on CoO_2_@SNHCT are presented in Figure S21.

**FIGURE 6:**
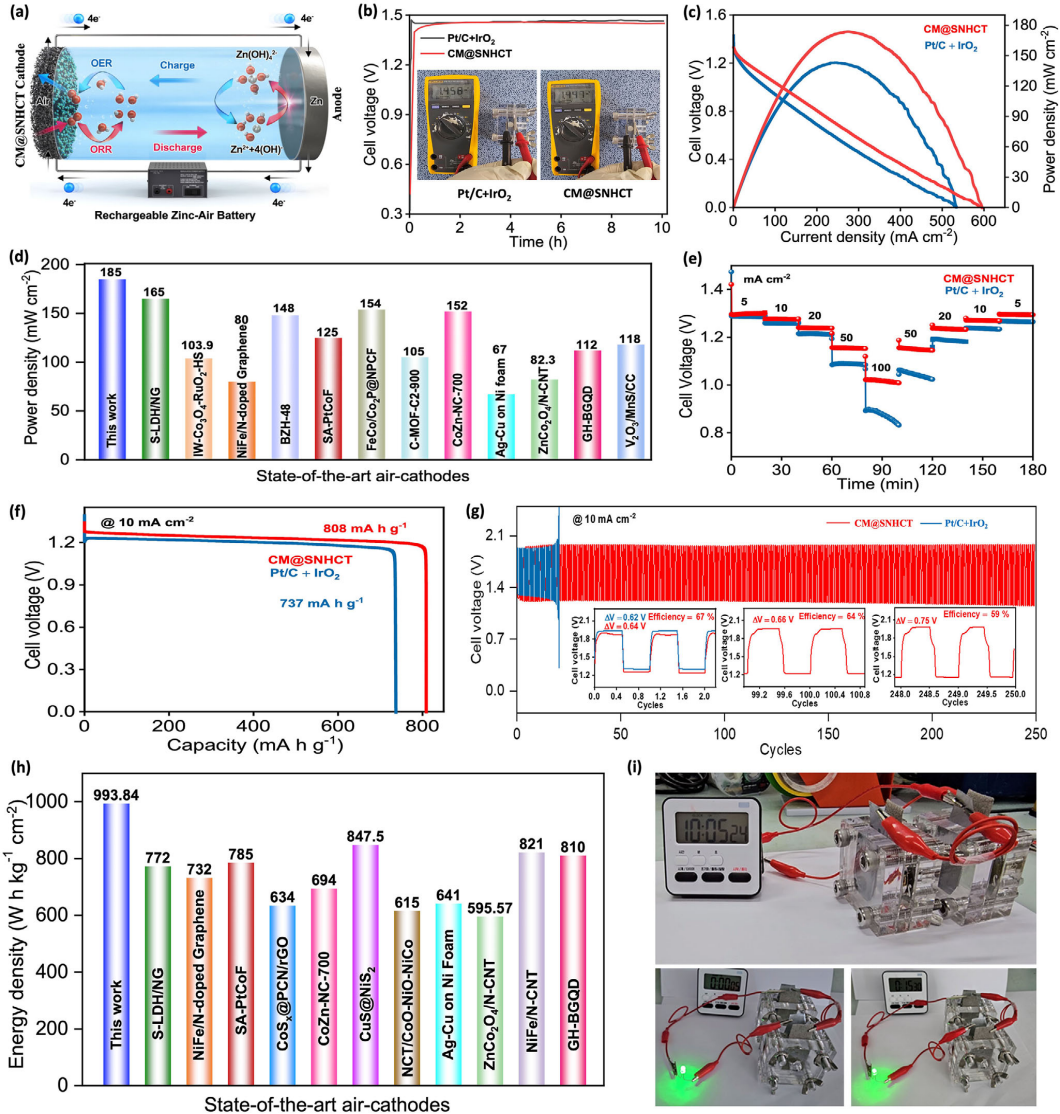
ZAB performance characterizations with CM@SNHCT cathode. (a) Schematic of the full‐cell ZAB, (b) open circuit voltage values of the ZABs with CM@SNHCT and Pt/C+IrO_2,_ and (c) their discharge polarization curves. (d) Peak‐power densities of the ZABs with CM@SNHCT (this work) and recently reported cathode materials. (e) Discharge rate capability performance of the ZABs with CM@SNHCT and Pt/C+IrO_2_, and their performance for (f) specific capacity and (g) cycling performance_._ (h) Energy densities of the ZABs with this work and state‐of‐the‐art other S‐LDH/NG, [30] NiFe/N‐doped Graphene, [31] SA‐PtCoF, [32] CoS+@PCN/rGO, [33] CoZn‐NC‐700, [34] CuS@NiS_2_, [35] NCT/CoO‐NiO‐NiCo, [36] Ag‐Cu on Ni Form, [37] ZnCO_2_O_4_/N‐CNT, [38] NiFe/N‐CNT, [39] and GH‐BGQD [40]. (i) Two ZAB full cells connected in series to power a digital timer and a green light‐emitting diode bulb.

The practical feasibility of CM@SNHCT as the air‐cathode was also evaluated by assembling a rechargeable ZAB, in which CM@SNHCT was employed as the air cathode and metallic Zn foil served as the anode, as schematically illustrated in Figure 6a. The open‐circuit voltage (OCV) profile of the assembled full cell is presented in Figure 6b. The CM@SNHCT‐based ZAB exhibits a stable OCV of 1.45 V over a continuous period of 10 h, which is nearly identical to that of the benchmark Pt/C+IrO_2_‐based ZAB (1.46 V). This high and stable OCV indicates favorable thermodynamic reversibility of the oxygen electrochemistry and minimal parasitic side reactions at the air cathode–electrolyte interface. The discharge polarization and corresponding power‐density curves are shown in Figure 6c. The CM@SNHCT‐based ZAB delivers a maximum peak power density of 185 mW·cm^−^^2^, which not only surpasses that of the Pt/C + IrO_2_‐based ZAB (170 mW·cm^−^^2^) but also exceeds the performance of recently reported state‐of‐the‐art ZAB cathode materials summarized in Figure 6d. This enhanced power output reflects the superior bifunctional ORR/OER kinetics and rapid mass transport enabled by the hierarchical porous architecture and optimized electronic structure of CM@SNHCT. To further assess rate capability, the discharge and charge performances were evaluated over a wide range of current densities from 5 to 100 mA·cm^−^^2^ (Figure 6e and Figure S22). The CM@SNHCT‐based ZAB maintains stable discharge and charge voltages at each applied current density, and notably, the voltage profile fully recovers when the current density is reduced from 100 mA·cm^−^^2^ back to 5 mA·cm^−^^2^. This behaviour demonstrates excellent electrochemical reversibility and structural robustness of the air cathode under dynamic operating conditions, which are critical for practical battery applications. The specific capacity of the ZAB, calculated based on the mass of consumed Zn, is presented in Figure 6f. At a discharge current density of 10 mA·cm^−^^2^, the CM@SNHCT‐based ZAB delivers a high specific capacity of 808 mAh·g^−^^1^, significantly outperforming the Pt/C+IrO_2_‐based ZAB (737 mAh·g^−^^1^) under identical conditions. This improvement indicates more efficient utilization of the Zn anode and reduced polarization losses during discharge, which can be attributed to the highly active and stable air cathode. Moreover, long‐term cycling stability was systematically evaluated by continuous galvanostatic charge–discharge tests conducted at a current density of 10 mA cm^−^^2^, with a fixed cycle duration of 30 min for both charging and discharging (Figure 6g). The CM@SNHCT‐based ZAB exhibits only a marginal increase in the charge–discharge voltage gap after 250 consecutive cycles, indicating excellent electrochemical reversibility and long‐term cycling durability. Notably, the discharge and charge plateaus remain highly stable throughout prolonged operation, reflecting suppressed polarization and minimal degradation of the air cathode. In contrast, the benchmark ZAB employing a Pt/C cathode for ORR and an IrO_2_ cathode for OER displays rapid performance decay, characterized by pronounced voltage polarization and a substantially widened charge–discharge gap within only 20 cycles. Furthermore, to comprehensively evaluate the electrochemical performance under different application scenarios, the electrolyte was replenished, and cycling stability tests were performed at various current densities. The CM@SNHCT‐based ZAB shows no significant increase in the voltage gap, withstanding over 1000 cycles at 10 mA cm^–^^2^, 450 cycles at 20 mA cm^–^^2^, and 300 cycles at 50 mA cm^–^^2^, as shown in Figure S23. This stark performance divergence signals the intrinsic limitations of conventional noble metal‐based catalysts under repeated ORR/OER cycling, particularly in terms of structural stability and resistance to interfacial degradation. The exceptional durability and reversibility of the CM@SNHCT‐based ZAB can be attributed to the rationally designed air‐cathode architecture, which synergistically integrates a mesoporous, hydrophobic sulfur‐doped hollow carbon nanotube framework with a highly conductive graphitic nitrogen‐rich carbon matrix. The interconnected mesoporous channels promote rapid oxygen diffusion and efficient electrolyte penetration, while the hydrophobic carbon framework mitigates electrolyte flooding and carbon corrosion. Simultaneously, the graphitic N‐rich network ensures fast electron transport and preserves the mechanical and chemical integrity of the catalyst during repeated oxygen reduction and evolution reactions. Owing to this optimized electrode design, the CM@SNHCT‐based ZAB delivers a gravimetric energy density of 993.84 Wh kg^−^^1^, which approaches the theoretical limit of 1086 Wh kg^−^^1^ for ZABs. As summarized in Figure 6h and Table S5, the CM@SNHCT‐based ZAB achieves a rare combination of high energy density and superior cycling stability with minimal voltage decay over extended operation places, far exceeding state‐of‐the‐art other ZABs, including S‐LDH/NG [36], NiFe/N‐doped Graphene [37], SA‐PtCoF [38], CoSx@PCN/rGO [39], CoZn‐NC‐700 [40], CuS@NiS_2_ [41], NCT/CoO‐NiO‐NiCo [42], Ag‐Cu on Ni Form [43], ZnCO_2_O_4_/N‐CNT [44], NiFe/N‐CNT [45], and GH‐BGQD [46]. These results demonstrate the effectiveness of the CM@SNHCT air cathode in sustaining high energy output over prolonged charge–discharge cycles. To further demonstrate its practical feasibility, two CM@SNHCT‐based ZAB cells connected in series were successfully employed to power a digital timer and a green LED, as shown in Figure 6i. The stable illumination and continuous operation of the electronic devices confirm the capability of the CM@SNHCT‐based ZABs to deliver reliable and sustained power under realistic working conditions. These findings establish CM@SNHCT‐based ZABs as promising candidates for high‐performance and durable next‐generation electrochemical energy‐storage technologies.

## 3 Conclusions

In summary, CM@SNHCT was rationally designed through a new synthetic strategy that combines in situ sulfidation with Zn/melamine evaporation of polydopamine‐coated Mn–Co/Zn MOFs grown on MTCA nanorods, enabling the formation of vacancy‐rich Co_9_S_6_._29_ precatalysts via ultrafine MnS‐induced lattice distortion within the SNHCT framework. During sulfidation, electron‐withdrawing sulfur species modulate the charge density of coordinatively unsaturated Co and Mn sites inherited from the MOF precursor, thereby optimizing the adsorption and desorption energetics of oxygen reaction intermediates and enhancing bifunctional ORR/OER activity. Mechanistic studies reveal the active involvement of lattice oxygen during OER. TMAOH probing confirms the operation of the LOM, while *operando* Raman spectroscopy and ^1^ ^8^O‐labeled DEMS analyses demonstrate the electrochemical reconstruction of Co_9_S_6_._29_ precatalysts into catalytically active CoOOH/CoO phases. This reconstruction is accompanied by reversible lattice oxygen‐vacancy formation and healing, which sustains high catalytic activity while suppressing irreversible overoxidation. DFT calculations further show that the reconstructed CoOOH/CoO@SNHCT structures exhibit reduced Gibbs free‐energy barriers for oxygen‐intermediate adsorption and conversion, while the strongly negative formation energy of CM@SNHCT indicates high thermodynamic stability. As a result, CM@SNHCT delivers a diffusion‐limited ORR current density surpassing commercial Pt/C, lower OER overpotentials than IrO_2_, and excellent electrochemical stability over 5000 cycles. At the device level, a ZAB assembled with CM@SNHCT as the air cathode and Zn metal as the anode achieves a near‐theoretical specific energy density of 993.8 Wh kg^−^^1^ while maintaining stable voltages over 250 cycles. Overall, this work demonstrates that the synergistic integration of defect modulation, controlled phase reconstruction, and self‐regulated lattice oxygen‐vacancy dynamics within hierarchically structured carbon frameworks enables highly efficient and durable bifunctional oxygen electrocatalysis, providing a viable design strategy for high‐performance ZABs and offering insights applicable to a broad range of electrochemical energy technologies.

## Author Contributions

**Dong Won Kim**: investigation, validation, formal analysis, visualization, writing – original draft, writing – review and editing. **Jong Hui Choi**: investigation, validation, formal analysis, visualization, writing – original draft, writing – review and editing. **Debarani Devi Khumujam**: conceptualization, investigation, validation, formal analysis, data curation, visualization, writing – original draft, writing – review and editing. **Ram Babu Ghising**: investigation, validation, formal analysis, visualization, writing – original draft, writing – review and editing. **Sang–Il Choi**: supervision, writing – review and editing. **Gwanho Lee**: investigation, validation, formal analysis, visualization, writing – original draft, writing – review and editing. **Do Hwan Kim**: supervision, writing – review and editing. **Jeung Ku Kang**: writing – original draft, funding acquisition, supervision, project administration, writing – review and editing. **Hyung Mo Jeong**: supervision, writing – review and editing. **Benzhi Wang**: investigation, validation, formal analysis, visualization, writing – original draft, writing – review and editing. **Saleem Sidra**: investigation, validation, formal analysis, visualization, writing – original draft, writing – review and editing.

## Conflicts of Interest

The authors declare no conflicts of interest.

## Supporting information




**Supporting File**: advs76208‐sup‐0001‐SuppMat.docx.

## Data Availability

The data that support the findings of this study are available from the corresponding author upon reasonable request.
