# Supplementary material for: Reconstruction of Ultrafine MnS‐Induced Vacancy‐Rich Co9S6.29 Precatalysts in Mesoporous S‐Doped N‐Rich Hollow Carbon Nanotubes Enables Dynamic O‐Vacancy Cycling for High‐Performance Zn‐Air Batteries
Source: Adv Sci (Weinh). 2026 Jun 26:e76208. Online ahead of print. doi: 10.1002/advs.76208 (PMC13336939; doi:10.1002/advs.76208)
Supplement: Supplementary file 1 — Supporting File: advs76208‐sup‐0001‐SuppMat.docx. [file ADVS-9999-e76208-s001.docx]

Supporting Information for

**Reconstruction of Ultrafine MnS-Induced Vacancy-Rich Co_9_S_6.29_ Precatalysts in Mesoporous S-Doped N-Rich Hollow Carbon Nanotubes Enables Dynamic O-Vacancy Cycling for High-Performance Zn-Air Batteries**

Debarani Devi Khumujam ^1^ | Saleem Sidra ^2^ | Ram Babu Ghising ^1^ | Dong Won Kim ^1^ | Jong Hui Choi ^1,3^ | Gwanho Lee ^4^ | Benzhi Wang ^5^ | Hyung Mo Jeong ^5^ | Sang-Il Choi ^4^ | Do Hwan Kim ^2,*^ | Jeung Ku Kang ^1,*^

^1^ Department of Materials Science and Engineering and NanoCentury Institute, Korea Advanced Institute of Science and Technology (KAIST), Daejeon, Republic of Korea

^2^ Department of Energy Storage/Conversion Engineering, Jeonbuk National University, Jeonju, Republic of Korea

^3^ School of Materials Science and Engineering, Yeungnam University, Gyeongsan, Republic of Korea

^4^ Department of Chemistry and Green-Nano Materials Research Center, Kyungpook National University, Daegu, Republic of Korea

^5^ Department of Smart Fab. Technology , Sungkyunkwan University, Suwon, Republic of Korea

^*^Corresponding authors: dhk201@jbnu.ac.kr, jeungku@kaist.ac.kr

**Table of Contents**

**S1.** **Experimental methods**

**S2.** **Supplementary figures and tables**

**S3.** **Supplementary references**

**S1. Experimental methods**

**S1.1. Synthesis**

**Synthesis of a melamine-thiocyanurate (MTCA) nanorod:** Melamine (1.94 g) was added to 70 mL of deionized water in an ice bath and stirred until fully dissolved. Separately, trithiocyanuric acid (2.73 g) was dissolved in 30 mL of deionized water. The two solutions were then combined and stirred for 20 min, after which the resulting precipitate was collected, washed three times with deionized water and ethanol, and dried at 60 °C for 12 h.

**Synthesis of Mn-incorporated-Co/Zn metal-organic frameworks on a MTCA (Mn-Zn/Co-MOF@MTCA):** MTCA (300 mg) was dispersed in 40 mL of a 1:1 (v/v) methanol–ethanol mixture, followed by the addition of 2-methylimidazole (16 mmol). Separately, Co(NO₃)₂ (1 mmol) and Zn(NO₃)₂ (0.5 mmol) were dissolved in 40 mL of the same methanol–ethanol mixture. The metal precursor solution was then added to the MTCA/2-methylimidazole dispersion and stirred for 2 min. The resulting mixture was aged at room temperature for 12 h without disturbance. The obtained Zn/Co-MOF@MTCA green precipitate was collected by centrifugation and dried at 60 °C for 12 h. To incorporate Mn, the Zn/Co-MOF@MTCA powder was dispersed in 40 mL ethanol, while the Mn salt was dissolved in 5 mL deionized water and added dropwise under stirring. The mixture was stirred for 24 h, washed three times with deionized water and ethanol, and dried in an oven overnight.

**Coating of Mn-ZnCo-MOF@MTCA with polydopamine:** **Mn–Zn/Co-MOF@MTCA (300 mg)** was dispersed in 30 mL of Tris buffer by sonication for 10 min. **Dopamine hydrochloride (35 mg)** was then added, and the mixture was stirred for 2 h to allow polydopamine (PDA) deposition. The resulting product was collected by centrifugation, washed three times, and dried in an oven for 12 h.

**Synthesis of ultrafine vacancy-rich Co sulfide-Mn sulfide heterostructures in a mesoporous hydrophobic S-doped N-rich conductive hollow carbon tube (CM@SNHCT):** The PDA-coated Mn–Co/Zn-MOF@MTCA powder was pyrolyzed at 900 °C for 2 h under an argon atmosphere to yield CM@SNHCT. The resulting product was subsequently subjected to acid leaching in 0.1 M HCl at 80 °C to remove unreacted and unstable metal species. For comparison, C@SNHCT, M@SNHCT, and CM were synthesized following the same procedure, except that C@SNHCT and M@SNHCT were prepared without cobalt and manganese precursors, respectively, and CM was synthesized without PDA coatings.

**S1.2. Material Characterizations**

The morphology of the prepared materials was characterized by field-emission scanning electron microscopy (FE-SEM, JSM-IT800) and transmission electron microscopy (TEM, Talos F200X G2). Elemental compositions were analyzed using energy-dispersive X-ray spectroscopy (EDS) attached to the TEM. Crystal structures and phase compositions were determined by X-ray diffraction (XRD, SmartLab, Rigaku) using Cu Kα radiation (λ = 1.5406 Å) operated at 40 kV and 30 mA. Raman spectra were collected using a Raman spectrometer (ARAMIS, Horiba Jobin Yvon). Surface chemical composition and elemental states were investigated by X-ray photoelectron spectroscopy (XPS, Axis Supra, Kratos). Nitrogen adsorption–desorption isotherms were measured at 77 K using a Micromeritics 3Flex analyzer to determine the Brunauer–Emmett–Teller (BET) specific surface area and pore size distribution. X-ray absorption fine structure (XAFS) measurements were performed at the XAFS (A1) beamline (R-XAS) at the Korea Institute of Science and Technology (KIST), Seoul, South Korea. X-ray absorption near-edge structure (XANES) and extended X-ray absorption fine structure (EXAFS) data were analyzed using the Athena and Artemis modules of the IFEFFIT software package.

**S1.3. Electrochemical Characterizations**

Electrochemical measurements were performed using a BioLogic VSP potentiostat–galvanostat workstation. Oxygen reduction reaction (ORR) activity was evaluated in a three-electrode configuration using O₂-saturated 0.1 M KOH as the electrolyte. A rotating disk electrode (RDE, 3 mm diameter) coated with catalyst ink served as the working electrode, while a Hg/HgO electrode and a carbon rod were used as the reference and counter electrodes, respectively. Catalyst ink was prepared by dispersing 5 mg of the active material in 1 mL ethanol containing 50 μL of 5 wt% Nafion solution, followed by sonication for 30 min. The ink was then drop-cast onto the RDE, and a fixed catalyst mass loading was maintained for all measurements. For comparison, commercial Pt/C (20 wt%) was tested under identical conditions. Polarization curves were recorded at a scan rate of 5 mV s⁻¹ and a rotation speed of 1600 rpm. All measured potentials were converted to the reversible hydrogen electrode (RHE) scale according to the following equation of

$E_{RHE}=E_{measured}+E_{Hg/HgO}^{0}+0.0592\times pH$ (S1)

with E°_Hg/HgO_= 0.098 V. To **determine the electron transfer number, polarization curves were recorded at various rotation speeds ranging from 400 to 3200 rpm.** The resulting RDE data were analyzed using the **Koutecky–Levich (K–L) equation,** and the number of electrons transferred (n) was calculated according to the following equations^[1]^:

$\frac{1}{j}=\frac{1}{j_{k}}+\frac{1}{j_{L}}=\frac{1}{j_{k}}+\frac{1}{B\sqrt{\omega}}$ (S2)

${B= 0.62\times D}_{O_{2}}^{2/3}\times C_{O_{2}}\times\nu^{-1/6}\times n\times F$ (S3)

where *j,* $j_{k}$, and $j_{L}$ are the measured, kinetic and diffusion limiting current densities, respectively, B is a constant, ω is the angular velocity of the RDE, D_O2_ is the oxygen diffusion coefficient (1.9×10^−5^ cm^2^·s ^−1^ ), C_O2_ is the bulk oxygen concentration (1.2×10^−6^ mol·cm^−3^ ), ν (0.01 cm^2^·s ^−1^) is the electrolyte viscosity, n is the electron transfer number, and F is the Faraday constant (96 485 C mol^−1^). Oxygen evolution reaction (OER) measurements were performed in 1.0 M KOH with standard three-electrode system. Polarization curves were recorded at a scan rate of 2 mV s⁻¹ with 95% *iR* compensation in which *i* is the current and *R* is the series resistance. The OER overpotential (η) was calculated as η = E_(RHE)_ − 1.23 V. Tafel slopes were derived from the polarization curves by fitting the linear region of the corresponding Tafel plots, as writted by

η = a + blog j (S4)

where η indicates the overpotential, b indicates the Tafel slope, and j indicates the current density. Additionally, the electric double layer capacitance (C_dl_) and electrochemically active surface area (ECSA) were determined from cyclic voltammogram (CV) measurements at scan rates ranging from 5 to 100 mV·s⁻¹, by the equation of

ECSA = C_dl_/C_s_ (S5)

where C_s_ is the specific capacitance. *Operando* Raman spectroscopy was performed using a chronoamperometric method. Electrochemical measurements were conducted by holding each potential for 5 min prior to spectral acquisition using an XperRAM-S567 spectrometer equipped with a 532 nm laser. The Raman exposure time was 20 s, and spectra were collected three times at each potential; the reported spectra represent the averaged data. *Operando* differential electrochemical mass spectrometry (DEMS) measurements were carried out using a quadrupole mass spectrometer (HPR-40, Hiden Analytical, UK) coupled with a Type-A electrochemical cell. Prior to measurements, the electrolyte was purged with high-purity Ar to remove dissolved oxygen. A three-electrode configuration was employed, consisting of a catalyst-coated glassy carbon disk (5 mm diameter) as the working electrode, a Hg/HgO reference electrode, and a Pt wire counter electrode. Catalyst ink was prepared by dispersing 10.0 mg of CM@SNHCTs or C@SNHCTs in 1.00 mL isopropanol containing 40 μL of 5 wt% Fumion FAA-3-SOLUT-10 ionomer, followed by drop-casting onto the working electrode and drying prior to use, yielding a catalyst loading of 2.0 mg cm⁻². For isotope-labeling experiments, the working electrode was cycled 50 times between 1.25 and 1.69 V vs. RHE at a scan rate of 5 mV s⁻¹ in 1.0 M KOH containing H₂¹⁸O (98 atom %, QuTope Inc.) to promote lattice-oxygen exchange. The electrode was then immersed in the same H₂¹⁸O-containing electrolyte for an additional 3 h to ensure complete labeling. After labeling, the electrode was thoroughly rinsed with H₂¹⁶O to remove physically adsorbed H₂¹⁸O and dried in a vacuum oven at 60 °C for 3 h. Subsequently, DEMS measurements were performed in N₂-saturated 1.0 M KOH containing H₂¹⁸O at an electrolyte flow rate of 5 μL s⁻¹. Cyclic voltammetry was conducted for three cycles between 1.25 and 1.75 V vs. RHE at 5 mV s⁻¹, while monitoring m/z = 32 (¹⁶O₂) and m/z = 34 (¹⁶O¹⁸O) signals. All experiments were repeated at least three times to ensure reproducibility.

**S1.4. Assembly and Configuration of Zn-air batteries (ZABs)**

An aqueous ZAB was assembled using a polished Zn foil (250 μm thick) as the anode and a catalyst-coated air cathode prepared by drop-casting the catalyst ink onto carbon paper with a polytetrafluoroethylene (PTFE) gas diffusion layer (Sigracet 39BB). The electrolyte consisted of 6.0 M KOH containing 0.2 M zinc acetate dihydrate [Zn(OAc)₂·2H₂O]. Pre-washed nickel foam was used as the current collector and backing layer, providing mechanical support and facilitating efficient transport of atmospheric O₂ to the gas diffusion layer. For comparison, a reference air cathode was fabricated using a mixture of commercial 20 wt% Pt/C and IrO₂ in a 1:1 mass ratio. The catalyst mass loading was kept constant for all cathodes.

**S1.5. Density Funtional Theory (DFT) Calculations**

**Density functional theory (DFT) calculations were performed using the Vienna** Ab initio **Simulation Package (VASP), with structural parameters informed by experimental XRD results.** The interaction between ion cores and valence electrons was described using the **projector augmented wave (PAW)** method. Exchange–correlation effects were treated within the **generalized gradient approximation (GGA)** using the **Perdew–Burke–Ernzerhof (PBE)** functional, with **Grimme’s DFT-D3** correction applied to account for long-range van der Waals interactions.^[2-4]^ Both Co sulfide and Mn sulfide were modeled with cubic crystal structures, exposing the (311) and (200) facets, respectively, consistent with JCPDS 03-065-1765 (Co sulfide) and 03-006-0518 (Mn sulfide). A 2 × 2 × 1 supercell of Mn sulfide was constructed as the starting model. The heterostructures were built using slab models with a 25 Å vacuum layer, with the surface slab positioned at the center to avoid spurious interactions. A S- and N-doped carbon layer was subsequently introduced beneath the CM heterostructure to simulate the experimental configuration. **Electronic structure analyses,** including the **density of states (DOS)** and **projected density of states (PDOS),** as well as **OER/ORR adsorption and binding energy calculations,** were carried out based on the optimized structures. A **plane-wave energy cutoff of 520 eV** was employed for all calculations. Structural optimization was performed until the **Hellmann–Feynman forces on each atom were below 0.02 eV Å⁻¹**. For total energy and DOS/PDOS calculations, **k-point meshes were generated using the VASPKIT package**.^[5]^ The **formation energies** were calculated according to the following equation:

E_F_ = E* - Σ*i* n*i* E_i_ (S6)

where E* represents the energy of each crystal structure, n_i_ denotes the number of elements in the crystal, and Ei indicates the energy of each individual element.

**Gibbs Free energy profiles for OER and ORR**

We further investigated the four-step reaction mechanisms for OER/ORR at various active sites under alkaline conditions. Under alkaline media, the four-electron transfer pathway^[6]^ for OER proceeds through the following elementary steps of

* + OH^−^  → OH* + e^-^  ∆G_1_ (S7-1)

OH* + OH^−^  → O*+ H_2_O + e^−^ ∆G_2_  (S7-2)

O* + OH^−^ → OOH* + e^-^  ∆G_3_ (S7-3)

OOH* + OH^−^ → O_2_ + * + H_2_O + e^−^  ∆G_4_ (S7-4)

where OH*, O*, and OOH* refer to the adsorption sites on the electrocatalyst surface. The Gibbs free energies were also computed for the following steps of

* + O_2_(g) + H_2_O(l) + e^-^ → OOH* + OH^-^ ∆G_1_  (S8-1)

OOH* + e^-^ → O* + OH^-^  ∆G_2_  (S8-2)

O* + H_2_O(l) + e^-^ → OH* + OH^-^ ∆G_3_ (S8-3)

OH* + e^-^ → * + OH^-^ ∆G_4_ (S8-4)

in which ΔG_1_, ∆G_2_, ΔG_3_, and, ∆G_4_ represent the Gibbes free energy changes for the four steps involving in OER and ORR. Moreover, the theoretical overpotential (η) values for OER and ORR were calculated using the following formula of

$\eta OER=\frac{\max\left\{ {\Delta G}_{1}, {\Delta G}_{2}, {\Delta G}_{3}, {\Delta G}_{4} \right\}}{e}-1.23 \left( V \right)$ (S9-1)

$\eta_{\mathrm{ORR}}=1.23 \left( V \right)-\frac{\min\left\{ {\Delta G}_{1}, {\Delta G}_{2}, {\Delta G}_{3}, {\Delta G}_{4} \right\}}{e}$ (S9-2)

**S2. Supporting figures and tables**

**Figure S1.** **Field emission scanning electron microscope (FE-SEM) images.** (a) melamine-thiocyanurate (MTCA) rod and (b) C@SNHCT.

**Figure S2. Morphological characterizations.** (a) Transmission electron microscopy (TEM) image, (b) high-angle annular dark field-scanning TEM (HAADF-STEM) image, (c) EDS color mapping images of CM.

**Figure S3.** **Brunauer-Emmett-Teller (BET) surface area and Barrett-Joyner-Halenda (BJH) pore size analyses.** (a) N_2_ adsorption-desorption isotherms and (b) pore size distributions of M@SNHCT, C@SNHCT, CM, CM@SNHCT, and SNHCT.

**Figure S4**. **X-ray photoelectron spectroscopy (XPS) survey spectra.** (a) XPS survey spectra, high-resolution (b) C1s spectra, (c) N1s spectra, and (d) S2p spectra of M@SNHCT, C@SNHCT, and CM@SNHCT.


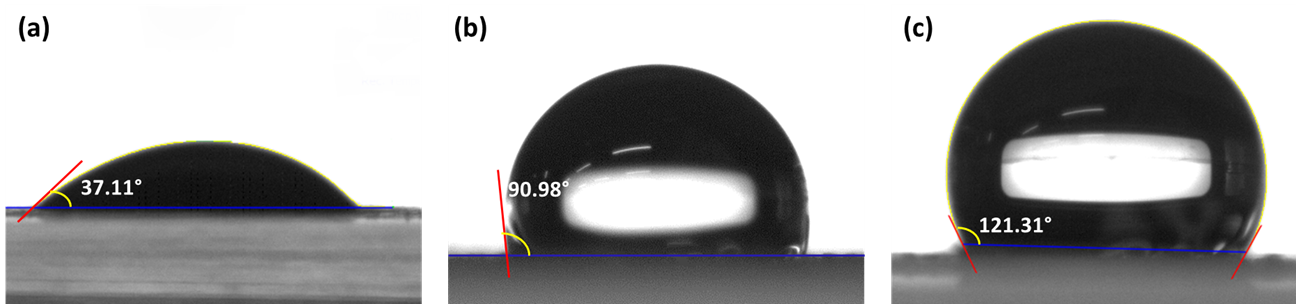


**Figure S5. Water contact angle measurements.** (a) Co-N/C, (b) C@SNHCT, and (c) CM@SNHCT.

**Figure S6. Wavelet transform (WT) X-ray absorption fine structure (EXAFS) contour plots.** (a) Co-foil and CoO, (b) Mn-foil and MnO.

**Figure S7**. **Cyclic voltammogram (CV) analysis and methanol tolerance test.** (a) CV curves measured at O_2_ and N_2_-saturated electrolyte for M@SNHCT and (b) methanol tolerance test results of CM@SNHCT and Pt/C.

**Figure S8. Linear sweep voltammetry (LSV) curves at different rotation speeds and corresponding electron transfer number Koutecky-Levich (K-L) plots**. (a-b) M@SNHCT, (c-d) C@SNHCT, (e-f) CM, and (g-h) CM@SNHCT.

**Fig. S9. Tafel plots of M@SNHCT, CM, C@SNHCT, CM@SNHCT, and IrO_2_ for OER.**

**Figure S10. CV curves with varying scan rates from 10 to 100 mV·s-1.** (a) M@SNHCT, (b) C@SNHCT, (c) CM, (d) CM@SNHCT, and (e) IrO_2_. (f) Double-layer capacitance (C_dl_) measurements for all synthesized electrocatalysts during OER.

**Figure S11. *Operando* Raman spectra of (a) C@SNHCT and (b) CM@SNHCT during OER process.**

**Fig. S12. OER LSV at different pH and TMAOH.** (a) C@SNHCT, (b) CM@SNHCT during OER process.

**Figure S13. Side and bottom view of optimized crystal structures.** (a) Co_9_S_2.69_, (b) MnS, (c) C@SNHCT, (d) M@SNHCT, (e) Co_9_S_2.69_/MnS, and (f) CM@SNHCT with 25Å vacuum along z-direction

**Figure S14. Projected density of states (PDOS) for d orbital of Co and Mn, p orbital of C, S, and N.** (a) Co_9_S_2.69_/MnS and (b) M@SNHCT.

**Figure S15. Calculated free energy diagrams for oxygen evolution reaction (OER).** (a) CM@SNHCT and (b) Co_9_S_2.69_/MnS at various active sites.

**Figure S16. Calculated free energy diagrams for oxygen reduction reaction (ORR).** (a) CM@SNHCT and (b) Co_9_S_2.69_/MnS at various active sites.

**Figure S17. Restructured models incorporating oxygen-vacancy defects.** (a) CoOOH/CoO@SNHCT and (b) CoO₂@SNCHT.

**Figure S18. Partial Density of States (PDOS)**. (a-b) CoOOH/CoO@SNHCT, showing the orbital contributions from cobalt (Co) atoms, including s, p, and d orbitals for both spin-up and spin-down states, and oxygen (O) atoms with separate spin-up and spin-down states for p_x_, p_y_, and p_z_ orbitals. (c-d) CoO_2_@SNHCT, focusing on the Co orbital contributions, including s, p, and d orbitals for both spin-up and spin-down states, and O atoms with separate spin-up and spin-down states for the oxygen p_x_, p_y_, and p_z_ orbitals.

**Figure S19. Gibbs free energy profiles for OER on MnO@SNHCT at the Mn active site.** (a) adsorbate evolution mechanism (AEM) and (b) lattice oxygen mechanism (LOM) pathways.

**
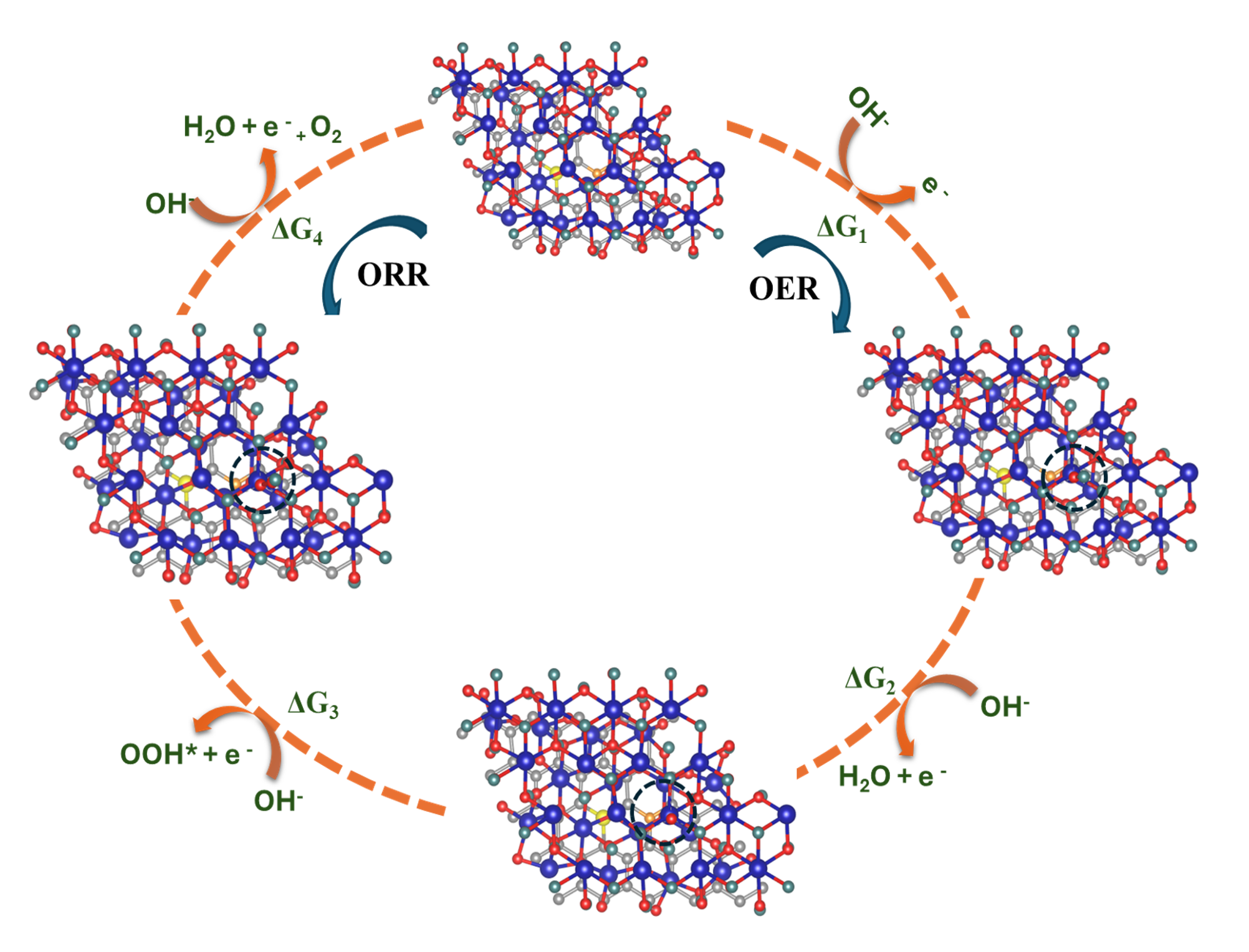
**

**Figure S20. Schematic illustration of the AEM for bifunctional ORR/OER catalysis on CoOOH/CoO@SNHCT, depicting stepwise formation of surface-adsorbed intermediates at the Co active site.**


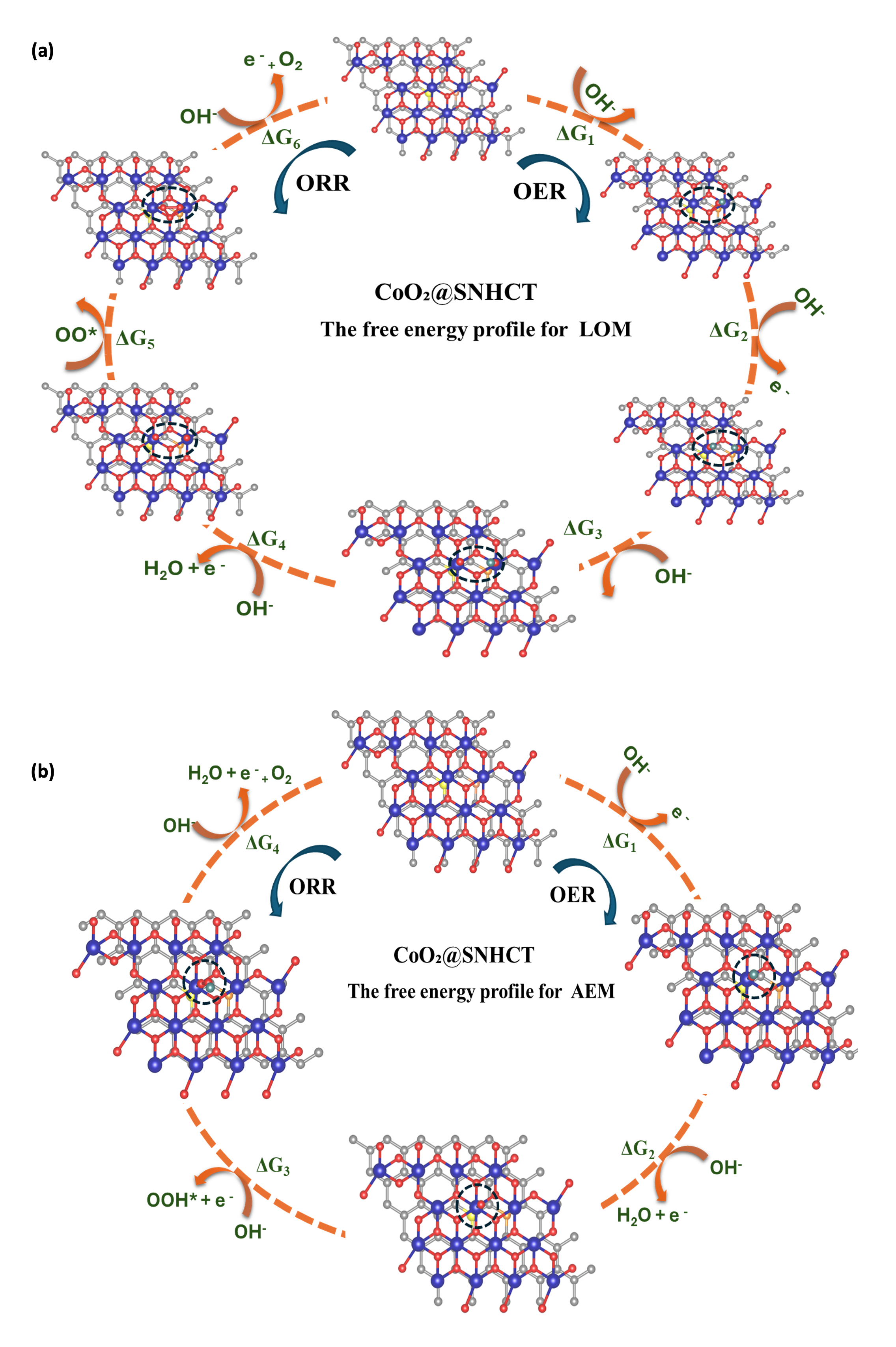


**Figure S21. Proposed schematic ORR/OER mechanisms for CoO₂@SNHCT electrocatalysts at the Co active site.** (a) LOM and (b) AEM pathways.

**Figure S22.** Charge rate capability performance of the ZAB with CM@SNHCT.

**Figure S23.** Cycling test of the CM@SNHCT-based ZAB at (a) 10 mA cm^–2^, (b) 20 mA cm^–2^, and (c) 50 mA cm^–2^.

**Table S1. Atomic and weight percentages of the constituent elements of CM@SNHCT obtained from TEM Energy-dispersive X-ray spectroscopy (TEM-EDS) analysis.**

| **Elements** | **Atomic %** | **Weight %** |
| --- | --- | --- |
| C | 62.63 | 31.65 |
| S | 12.79 | 17.25 |
| N | 5.17 | 3.04 |
| Co | 18.31 | 45.38 |
| Mn | 1.10 | 2.68 |

**Table S2. E_10_ /η_10,_ E_o_ , E_1/2,_ and △E values of CM@SNHCT and recently reported cathode materials for OER.**

| Cathode Material | E_10_ /η_10_  (V) | E_o_  (V) | E_1/2_  (V) | △E  (V) | Reference |
| --- | --- | --- | --- | --- | --- |
| CM@SNHCT | E_10_ **=** 1.50  η_10_ **=** 275 | 0.93 | 0.84 | 0.68 | This work |
| Co_9_S_8_@MoS_2_ | E_10_ **=** 1.57 | - | 0.844 | - | [7] |
| α/β-NiMoO_4_ | E_10_ **=** 1.54 | - | 0.72 | - | [8] |
| Mn/Co-N-C-0.02-800 | E_10_ **=** 1.66 | 0.90 | 0.80 | 0.86 | [9] |
| CoS_x_@Cu_2_MoS_4_-MoS_2_/NSG | E_10_ **=** 1.58 | 0.97 | 0.89 | 0.69 | [10] |
| S-CFZ | E_10_ **=** 1.52 | 0.85 | 0.82 | 0.68 | [11] |
| NiFe_2_O_4_/FeNi_2_S_4_ HNSs | η_10_ **=** 492 | 0.715 | 0.507 | 1.15 | [12] |
| CoNi-CoN_4_-HPC-900 | E_10_ **=** 1.70 | 1.01 | 0.78 | 0.90 | [13] |
| Co_9_S_8_/NSG‑700 | E_10_ **=** 1.61 | 0.92 | 0.79 | 0.82 | [14] |
| CuS/NiS_2_ | η_10_ **=** 290 | 0.89 | 0.84 | 0.79 | [15] |
| Fe,Co/DSA-NSC | η_10_ **=** 281 | - | 0.873 | 0.56 | [16] |

**Table S3. Gibbs free energy changes and overpotentials of Co_9_S_2.69_, MnS, C@SNHCT, M@SNHCT, Co_9_S_2.69_/MnS, and CM@SNHCT at various active sites for OER, as determined through density functional theory (DFT) calculations.**

| Cathode Material | Active  site | Change in Gibbs energy (eV) | | | | Overpotential  (η) |
| --- | --- | --- | --- | --- | --- | --- |
|  |  | ΔG_1_ | ΔG_2_ | ΔG_3_ | ΔG_4_ |  |
| Co_9_S_2.69_ | Co | 2.143 | 2.993 | 0.047 | -0.263 | 1.76 |
|  | S | 2.008 | 3.026 | 0.306 | -0.420 | 1.80 |
| MnS | Mn | 3.167 | 2.094 | -0.312 | -0.029 | 1.94 |
|  | S | 3.267 | 1.994 | -0.412 | 0.071 | 2.04 |
| C@SNHCT | Co | 1.726 | 1.347 | 0.701 | 1.146 | 0.50 |
|  | S | 1.745 | 1.326 | 0.606 | 1.243 | 0.51 |
| M@SNHCT | Mn | 1.358 | 1.203 | 0.335 | 2.024 | 0.79 |
|  | S | 1.266 | 1.394 | 0.088 | 2.172 | 0.94 |
| Co_9_S_2.69_/MnS | Co | 0.530 | 1.346 | 1.746 | 1.297 | 0.52 |
|  | S | 1.184 | 0.369 | 1.997 | 1.371 | 0.77 |
|  | Mn | 0.627 | 1.164 | 1.882 | 1.247 | 0.65 |
|  | S | 0.353 | 1.264 | 1.942 | 1.360 | 0.71 |
| CM@SNHCT | Co | 0.873 | 1.559 | 0.829 | 1.659 | 0.43 |
|  | S | 0.815 | 1.417 | 0.930 | 1.758 | 0.53 |
|  | Mn | 0.964 | 1.439 | 0.842 | 1.675 | 0.45 |
|  | S | 0.829 | 1.421 | 0.848 | 1.822 | 0.59 |

**Table S4. DFT Gibbs free energy changes and overpotentials of Co_9_S_2.69_, MnS, C@SNHCT, M@SNHCT, Co_9_S_2.69_/MnS, and CM@SNHCT at various active sites for ORR.**

| Cathode Material | Active  site | Change in Gibbs energy (eV) | | | | Overpotential  (η) |
| --- | --- | --- | --- | --- | --- | --- |
|  |  | ΔG_1_ | ΔG_2_ | ΔG_3_ | ΔG_4_ |  |
| Co_9_S_2.69_ | Co | -2.143 | -2.993 | -0.047 | 0.263 | 1.49 |
|  | S | -2.008 | -3.026 | -0.306 | 0.420 | 1.65 |
| MnS | Mn | -3.167 | -2.094 | 0.312 | 0.029 | 1.54 |
|  | S | -3.267 | -1.994 | 0.412 | -0.071 | 1.64 |
| C@SNHCT | Co | -1.726 | -1.347 | -0.701 | -1.146 | 0.53 |
|  | S | -1.745 | -1.326 | -0.606 | -1.243 | 0.62 |
| M@SNHCT | Mn | -1.358 | -1.203 | -0.335 | -2.024 | 0.89 |
|  | S | -1.266 | -1.394 | -0.088 | -2.172 | 1.14 |
| Co_9_S_2.69_/MnS | Co | -0.530 | -1.346 | -1.746 | -1.297 | 0.70 |
|  | S | -1.184 | -0.369 | -1.997 | -1.371 | 0.86 |
|  | Mn | -0.627 | -1.164 | -1.882 | -1.247 | 0.60 |
|  | S | -0.353 | -1.264 | -1.942 | -1.360 | 0.88 |
| CM@SNHCT | Co | -0.873 | -1.559 | -0.829 | -1.659 | 0.40 |
|  | S | -0.815 | -1.417 | -0.930 | -1.758 | 0.41 |
|  | Mn | -0.964 | -1.439 | -0.842 | -1.675 | 0.39 |
|  | S | -0.829 | -1.421 | -0.848 | -1.822 | 0.40 |

**Table S5. Electrochemical characterizations of ZABs with CM@SNHCT and recently reported air-cathodes.**

| Cathode Material | Open Curcuit Voltage (V) | | | | | Power Density  (mW ·cm^-2^) | | Specific Capacity (mAh·g^-1^@ mA ·cm^-2^) | | Energy Density  (Wh·kg^-1^@ mA ·cm^2^) | | Cycling  Performance | | Referenc |
| --- | --- | --- | --- | --- | --- | --- | --- | --- | --- | --- | --- | --- | --- | --- |
| CM@SNHCT | | 1.45 | | | | 185.0 | | 808@10 | | 993.84@10 | | 250 h, 250 cycles, initial △E_i_= 0.64, final △E_f_= 0.75 | | This work |
| S–LDH/NG | | 1.43 | | 165.0 | | | | 740@10 | | 772@5 | | 80 h, △E_i_= 0.68, △E_f_= 0.75 | | [17] |
| IW-Co_3_O_4_-RuO_2_-HS | | 1.50 | | 103.9 | | | | 759.5@10 | | - | | 100 h, △E_i_= 0.86, △E_f_= 0.94 | | [18] |
| NiFe/N-Doped  Graphene | | - | | ~80 | | | | 583.7@10 | | 732@10 | | 34 h, △E_i_= 0.39, △E_f_= 0.68 | | [19] |
| BHZ-48 | | 1.49 | | 148.0 | | | | - | | - | | 150 h, △E_i_= 0.80, △E_f_= 0.85 | | [20] |
| SA-PtCoF | | 1.30 | | 125.0 | | | | 808@10 | | 785@10 | | 240 h | | [21] |
| CoS_x_@PCN/rGO | | 1.38 | | - | | | | - | | 634 | | 43.8 h | | [22] |
| FeCo/Co_2_P@ NPCF | | 1.44 | | 154.0 | | | | - | | - | | 107 h, △E_i_= 0.83, △E_f_= 0.80 | | [23] |
| C-MOF-C2-900 | | 1.46 | | 105.0 | | | | 741@10 | | - | | 40 h | | [24] |
| CoZn-NC-700 | | 1.42 | | 152.0 | | | | 578@10 | | 694@10 | | 64 h, △E_i_= 0.73, △E_f_= 1.10 | | [25] |
| CuS/NiS_2_ | | 1.44 | | 172.4 | | | | 678@10 | | 847.5@10 | | 83 h; △E_i_= 0.57, △E_f_=negligible fading | | [15] |
| NCNT/Co  O-NiO-NiCo | | - | | - | | | | 545 @20 | | 615@20 | | - | | [26] |
| Ag-Cu on Nickle  Foam | | - | | 67.0 | | | | 572@20 | | 641@20 | | - | | [27] |
| ZnCo_2_O_4_/N-CNT | | - | | 82.3 | | | | 428.47@10 | | 595.57@10 | | - | | [28] |
| NiFe/N-CNT | | 1.48 | | 300.7 | | | | 739.9@20 | | 821@20 | | 100 h | | [29] |
| GH-BGQD | | 1.40 | | 112.0 | | | | 687 | | 810@10 | | 100 h | | [30] |
|  | | |  | |  | |  | |  | |  | |  | |

**S3. Supplementary References**

[1] A. J., Bard, L. R. Faulkner, *J. Wiley and Sons* 2000, **ISBN 0471043729**.

[2] S. Ehrlich, J. Moellmann, W. Reckien, T. Bredow, S. Grimme, *ChemPhysChem* 2011, **12**, 3414–3420.

[3] G. Kresse and J. Hafner, *J. Phys. Condens. Matter* 1994, **6**, 8245-8257.

[4] G. Kresse and D. Joubert, *Phys. Rev. B - Condens. Matter Mater. Phys.* 1999, **59**, 1758-1775.

[5] V. Wang, N. Xu, J. C. Liu, G. Tang and W. T. Geng, *Comput. Phys. Commun.* 2021, **267**, 108033.

[6] S. Ramakrishnan, D. B. Velusamy, S. Sengodan, G. Nagaraju, D. H. Kim and A. R. Kim, D. J. Yoo, *Appl. Catal. B Environ.* 2022, **300**, 120752.

[7] J. Bai, T. Meng, D. Guo, S. Wang, B. Mao and M. Cao, *ACS Appl. Mater. Interfaces*, 2018, **10**, 1678–1689.

[8] J. Zhu, Q. Zhou, L. Wang, W. Zhou, M. Chen, X. Liu, D. Gao, D. Chao, *Adv. Energy Mater.* 2024, **14**, 2304554.

[9] L. Wei, L. Qiu, Y. Liu, J. Zhang, D. Yuan and L. Wang, *ACS Sustain. Chem. Eng.*, 2019, **7**, 14180–14188.

[10] D. C. Nguyen, D. T. Tran, T. L. L. Doan, D. H. Kim, N. H. Kim and J. H. Lee, *Adv. Energy Mater.*, 2020, **10**, 190328.

[11] Y. Jiang, Y.-P. Deng, R. Liang, N. Chen, G. King, A. Yu and Z. Chen, *J. Am. Chem. Soc.*, 2022, **144**, 4783–4791.

[12] L. An, Z. Zhang, J. Feng, F. Lv, Y. Li, R. Wang, M. Lu, R. B. Gupta, P. Xi and S. Zhang, *J. Am. Chem. Soc.*, 2018, **140**, 17624–17631.

[13] Y. Liu, Z. Chen, Z. Li, N. Zhao, Y. Xie, Y. Du, J. Xuan, D. Xiong, J. Zhou, L. Cai and Y. Yang, *Nano Energy*, 2022, **99**, 107325.

[14] Q. Shao, J. Liu, Q. Wu, Q. Li, H. guo Wang, Y. Li and Q. Duan, *Nano-Micro Lett.*, 2019, **11**, 4.

[15] L. An, Y. Li, M. Luo, J. Yin, Y. Zhao, C. Xu, F. Cheng, Y. Yang, P. Xi and S. Guo, *Adv. Funct. Mater.*, 2017, **27**, 1703779.

[16] G. Yasin, S. Ali, S. Ibraheem, A. Kumar, M. Tabish, M. A. Mushtaq, S. Ajmal, M. Arif, M. A. Khan, A. Saad, L. Qiao and W. Zhao, *ACS Catal.*, 2023, **13**, 2313–2325.

[17] X. Han, N. Li, J. S. Baik, P. Xiong, Y. Kang, Q. Dou, Q. Liu, J. Y. Lee, C. S. Kim and H. S. Park, *Adv. Funct. Mater.*, 2023, **33**, 2212233.

[18] Y. Gao, D. Zheng, Q. Li, W. Xiao, T. Ma, Y. Fu, Z. Wu and L. Wang, *Adv. Funct. Mater.*, 2022, **32**, 2203206.

[19] J. Zhu, M. Xiao, Y. Zhang, Z. Jin, Z. Peng, C. Liu, S. Chen, J. Ge and W. Xing, *ACS Catal.*, 2016, **6**, 6335–6342.

[20] Y. Jiang, Y. P. Deng, R. Liang, J. Fu, R. Gao, D. Luo, Z. Bai, Y. Hu, A. Yu and Z. Chen, *Nat. Commun.*, 2020, **11**, 1166.

[21] Z. Li, W. Niu, Z. Yang, N. Zaman, W. Samarakoon, M. Wang, A. Kara, M. Lucero, M. V. Vyas, H. Cao, H. Zhou, G. E. Sterbinsky, Z. Feng, Y. Du and Y. Yang, *Energy Environ. Sci.*, 2020, **13**, 884–895.

[22] W. Niu, Z. Li, K. Marcus, L. Zhou, Y. Li, R. Ye, K. Liang and Y. Yang, *Adv. Energy Mater.*, 2018, **8**, 1–8.

[23] Q. Shi, Q. Liu, Y. Ma, Z. Fang, Z. Liang, G. Shao, B. Tang, W. Yang, L. Qin and X. Fang, *Adv. Energy Mater.*, 2020, **10**, 1903854.

[24] M. Zhang, Q. Dai, H. Zheng, M. Chen and L. Dai, *Adv. Mater.*, 2018, **30**, 1705431.

[25] B. Chen, X. He, F. Yin, H. Wang, D. Liu, R. Shi, J. Chen and H. Yin, *Adv. Funct. Mater.*, 2017, **27**, 1700795.

[26] X. Liu, M. Park, M. G. Kim, S. Gupta, G. Wu and J. Cho, *Angew. Chemie Int. Ed.*, 2015, **54**, 9654–9658.

[27] Y. Lei, F. Chen, Y. Jin and Z. Liu, *Nanoscale Res. Lett.*, 2015, **10**, 197.

[28] Z. Q. Liu, H. Cheng, N. Li, T. Y. Ma and Y. Z. Su, *Adv. Mater.*, 2016, **28**, 3777–3784.

[29] H. Lei, Z. Wang, F. Yang, X. Huang, J. Liu, Y. Liang, J. Xie, M. S. Javed, X. Lu, S. Tan and W. Mai, *Nano Energy*, 2020, **68**, 104293.

[30] T. Van Tam, S. G. Kang, M. H. Kim, S. G. Lee, S. H. Hur, J. S. Chung and W. M. Choi, *Adv. Energy Mater.*, 2019, **9**, 1900945.
